# Secondary Evolution of a Self-Incompatibility Locus in the Brassicaceae Genus Leavenworthia

**DOI:** 10.1371/journal.pbio.1001560

**Published:** 2013-05-14

**Authors:** Sier-Ching Chantha, Adam C. Herman, Adrian E. Platts, Xavier Vekemans, Daniel J. Schoen

**Affiliations:** 1 Department of Biology, McGill University, Montreal, Quebec, Canada; 2 Laboratoire de Génétique et Évolution des Populations Végétale, Unité Mixte de Recherche 8198, Centre National de Recherches Scientifiques–Université Lille 1, Sciences et Technologies, Cité Scientifique, Villeneuve d'Ascq, France; Cornell University, United States of America

## Abstract

Self-incompatibility (SI) is the flowering plant reproductive system in which self pollen tube growth is inhibited, thereby preventing self-fertilization. SI has evolved independently in several different flowering plant lineages. In all Brassicaceae species in which the molecular basis of SI has been investigated in detail, the product of the *S-locus receptor kinase* (*SRK*) gene functions as receptor in the initial step of the self pollen-rejection pathway, while that of the *S-locus cysteine-rich* (*SCR*) gene functions as ligand. Here we examine the hypothesis that the *S* locus in the Brassicaceae genus Leavenworthia is paralogous with the *S* locus previously characterized in other members of the family. We also test the hypothesis that self-compatibility in this group is based on disruption of the pollen ligand-producing gene. Sequence analysis of the *S*-locus genes in Leavenworthia, phylogeny of *S* alleles, gene expression patterns, and comparative genomics analyses provide support for both hypotheses. Of special interest are two genes located in a non-*S* locus genomic region of *Arabidopsis lyrata* that exhibit domain structures, sequences, and phylogenetic histories similar to those of the *S*-locus genes in Leavenworthia, and that also share synteny with these genes. These *A. lyrata* genes resemble those comprising the *A. lyrata S* locus, but they do not function in self-recognition. Moreover, they appear to belong to a lineage that diverged from the ancestral Brassicaceae *S*-locus genes before allelic diversification at the *S* locus. We hypothesize that there has been neo-functionalization of these *S*-locus-like genes in the Leavenworthia lineage, resulting in evolution of a separate ligand-receptor system of SI. Our results also provide support for theoretical models that predict that the least constrained pathway to the evolution of self-compatibility is one involving loss of pollen gene function.

## Abstract 2

Self-incompatibility enables plants to avoid inbreeding by self-pollination. Here we report that the genetic locus encoding self-pollen recognition has evolved twice in the Brassicaceae family, challenging the notion that loss of self-incompatibility is irreversible.

## Author Summary

Self-incompatibility (SI) is a pollen recognition system that enables plants to avoid the inbreeding caused by self-pollination. It involves a pair of tightly linked genes known as the *S* locus. The product of one of these genes acts as the receptor and recognizes the pollen protein produced by the same plant, while the product of the other gene is the pollen protein that is recognized by the receptor. In this study, we have analyzed the gene sequence, genome organization, and gene evolutionary history of *S* loci in members of the Brassicaceae family, which includes plants of the genus Leavenworthia. From our analyses, we conclude that both genes that comprise the ancestral *S* locus in the Brassicaceae were lost in Leavenworthia. We show, however, that plants of this genus possess two other linked genes that exhibit patterns of polymorphism and expression that are characteristic of an *S* locus. These genes occupy the same genomic position in Leavenworthia as do two non-*S*-locus genes in the related species *Arabidopsis lyrata*, genes that are not known to function in self-recognition in this species. We suggest that these genes have evolved to assume the function of the pollen recognition system of SI in Leavenworthia—that is, that there has been de novo emergence of a distinct Brassicaceae *S* locus in this genus. We also present evidence that the breakdown of the SI system in two Leavenworthia races is due to independent mutations in the *S*-locus pollen gene, in accordance with theoretical predictions for the spread of *S*-locus disrupting mutations.

## Introduction

Self-incompatibility (SI) is a widespread plant reproductive system that prevents inbreeding by facilitating the rejection of self-pollen. It is a major evolutionary feature of the flowering plants [1]. SI is a complex phenotype whose functioning requires co-evolution among several interacting components [2]. It has been proposed that SI evolved several times in the angiosperms [3], a hypothesis supported by molecular investigations that have also helped pinpoint the genes that control pollen specificity, pollen recognition, and the downstream reactions that mediate cessation of pollen tube growth [4]. The evolutionary loss of SI leading to self-compatibility (SC) and the potential for the shift to self-fertilization is often stated to be irreversible [5],[6].

Despite increasing knowledge of the mechanisms that underlie SI, the question remains as to how such a complex system could have evolved independently in many different angiosperm lineages. One answer may lie in the phenomenon of neo-functionalization of genes. It has been noted that the mechanisms that underlie SI share a number of features with another important plant function, namely pathogen recognition and rejection [7]. Moreover, it has become increasingly clear that evolution can reshuffle and reshape functions through exon recruitment and domain swapping [8], and so it is conceivable that SI could have evolved by co-opting genes with receptor and signaling roles that initially functioned in plant defense. Neo-functionalization of genes has been shown to be most likely when there are strong selection pressures [9]. The avoidance of inbreeding and its negative fitness consequences provide one such selective context [10].

In the sporophytic type of self-incompatibility (SSI), the pollen and stigma SI phenotypes (or “specificities”) are controlled by the diploid genotype of the parent (the sporophyte) [11]. SSI is known from 10 families of flowering plants [12]. It has been best characterized in the Brassicaceae family. In Arabidopsis and Brassica (and several other closely related Brassicaceae), the SI locus (*S* locus) contains two tightly linked genes that have been shown to be principally responsible for the SI phenotype [2],[11],[13],[14]. One of these genes, the *S*-locus receptor kinase (*SRK*), produces a transmembrane receptor expressed in the stigma. The extracellular domain of this protein can bind to the secreted protein ligand produced by the other *S*-locus gene, the *S*-locus cysteine-rich gene (*SCR*, also known as *SP11*), which is expressed in the tapetum of anthers, coating pollen with the protein product [15],[16]. When self-pollen recognition occurs, it initiates a signaling cascade that prevents self-pollen hydration and growth of the pollen tube [17],[18].

Though not included in the initial studies of the molecular basis of SSI in the Brassicaceae, the genus Leavenworthia has played an important role in evolutionary studies of plant mating systems. Detailed biosystematic work in the genus [19] documenting both inter- and intraspecific variation in the presence/absence of SI in a geographically localized region of the southern United States led to many subsequent investigations that focused especially on the ecology and population genetics of the group [20]–[24]. More recently, application of molecular genetic tools to the study of Leavenworthia uncovered a locus that co-segregates with the SI reaction, exhibits high levels of polymorphism, forms an allele phylogeny characterized by long terminal branches, and exhibits high effective rates of migration, and trans-specific polymorphism of alleles [25]–[28], all expected features for the *S* locus.

The portion of the Leavenworthia *S* locus sequenced in earlier studies contains a number of characteristics also reported for *SRK* in other Brassicaceae, in particular an exon sequence that is similar to that of the SRK extracellular domain (*S*-domain), which contains several hypervariable regions thought to be involved in pollen recognition [25]. This gene was referred to as *Lal2*. Despite published evidence that *Lal2* functions as *SRK* in Leavenworthia, the full sequence of the gene (i.e., the expected seven exons coding for the entire extracellular *S*-domain, transmembrane domain, and kinase domain) could not be PCR-amplified using primers anchored in conserved regions of the *SRK* coding sequence, and no *SCR* gene (which is expected to be present in the genome close to *SRK*) was detected using PCR-based approaches. Moreover, the bulk of *Lal2* alleles do not cluster phylogenetically with the *SRK* alleles of Arabidopsis, Brassica, and other Brassicaceae species. Two putative *S* alleles exhibiting sequence similarity to the *S*-domain of *Arabidopsis lyrata SRK* have been observed, but these represent fewer than 3% of the *Lal2* alleles characterized to date [25], and in a series of five separate diallel crosses involving 20 plants, *Lal2* allele sequences in each of 19 plants correctly predicted compatibility relationships, further indicating that it is unlikely that our investigations have failed to uncover the bulk of Leavenworthia *S*-locus haplotypes. The phylogenetic relationships of Leavenworthia *S* alleles to others in the Brassicaceae family is unexpected, especially given that biosystematic studies place the genus Leavenworthia in the tribe Cardamineae, which is more closely related to Arabidopsis and Capsella than to Brassica [29].

In this report we present new data on the Leavenworthia *S* locus gleaned from fosmid cloning, sequencing, expression analysis, comparative genomic, and crossing studies. While sequence characteristics and tissue expression pattern of both the pollen and stigma genes strongly support the hypothesis that the previously described *Lal2* gene forms a portion of the Leavenworthia *S* locus, comparative synteny studies, along with closer examination of sequence variation at this locus, suggest that the Arabidopsis *S*-locus ortholog was lost in Leavenworthia following the divergence of the group from the common ancestor with other members of the Cardamineae. In addition, phylogenetic analysis of *Lal2*, *SRK*, and other gene family members suggests that SI in this genus is based on genes that have diversified separately and are thus likely paralogous to Arabidopsis *SRK* and *SCR*. We also show that two separate losses of SI in one species of Leavenworthia (*L. alabamica*) are likely due to independent mutations in the *SCR*-like gene coding sequence and/or its promoter. Together these results portray SI as a reproductive system that is more evolutionarily plastic than previously believed.

## Results

### Fosmid and PCR Cloning of the Lal2 Region in Different Races of *Leavenworthia alabamica*

*Leavenworthia alabamica* includes several races that differ in floral characteristics and mating system [20]. The *L. alabamica* populations studied here belong to three races. The a1 race consists of SI plants with large, strongly scented flowers, and outwardly dehiscing anthers. Plants of race a2 are SC, with large but weakly scented flowers, and partially inward dehiscing anthers, while a4 plants are also SC, but with small flowers lacking scent, and fully inward dehiscing anthers.

To better characterize the *Leavenworthia alabamica Lal2* (*LaLal2*) gene and gain knowledge about its genomic context, fosmid libraries were constructed from single individuals of all three races. Clones containing *LaLal2* were isolated after screening the libraries by PCR, and their sequences were obtained using 454 sequencing technology. The a1 race plant was heterozygous at *LaLal2*, whereas the a2 and a4 race plants were each homozygous for different *LaLal2* alleles (whose *S*-domain sequences match those previously reported in these races [25]). One *LaLal2*-containing clone was obtained from each of the a1 race and a2 race libraries (35,750 bp and 39,236 bp, respectively). From the a4 race library, two overlapping clones were isolated; these assembled into one long contig of 64,895 bp. The assembled sequences from the different *L. alabamica* races cover a similar genomic region, and they share a number of structural features characteristic of other Brassicaceae *SRK/SCR S* loci. We therefore refer to them below as Leavenworthia *S* haplotypes. Also included in our analysis are partial sequences, obtained by PCR amplification, of an additional *S* haplotype found in a population of fully SI plants belonging to the a1 race. This *S* haplotype contains a *LaLal2 S*-domain sequence identical to that of the SC race a2. To distinguish between the a1 haplotype from the a1 fosmid clone and this second a1 haplotype, they are referred to below as a1-1 and a1-2, respectively.

### The *Leavenworthia alabamica Lal2* Gene Encodes a Putative Receptor Kinase That Shares Highest Homology with a Paralog of *SRK* in *A. lyrata*

Previous sequence information available for *LaLal2* was limited to the portion of the sequence corresponding to the extracellular domain of members of the *S*-domain 1 (SD-1) receptor-like kinase (RLK) gene family to which *SRK* belongs [25]. Analysis of the fosmid clones sequences allowed the full-length genomic sequence of *LaLal2* to be determined. Homology of the full-length genomic *LaLal2* sequence extends over the entire length expected for genes belonging to the SD-1 receptor kinase family. After excluding other Leavenworthia sequences, the highest match obtained from our BLASTn searches with the genomic *LaLal2* sequence was NCBI Gene ID 9305017 from *Arabidopsis lyrata* (coverage 41%, E value 2e-106), which has no characterized function (Table S1). For brevity the NCBI Gene ID 9305017 will be referred to as the *Arabidopsis lyrata Lal2* (*AlLal2*) gene. Other, lower similarity matches were to Brassicaceae *SRK* sequences. We determined the *LaLal2* coding regions by combining data obtained from RT-PCR and 5′/3′ RACE sequences, which show that the gene has seven exons (Figure S1A), as observed in *SRK* [30].

**Figure 1:**
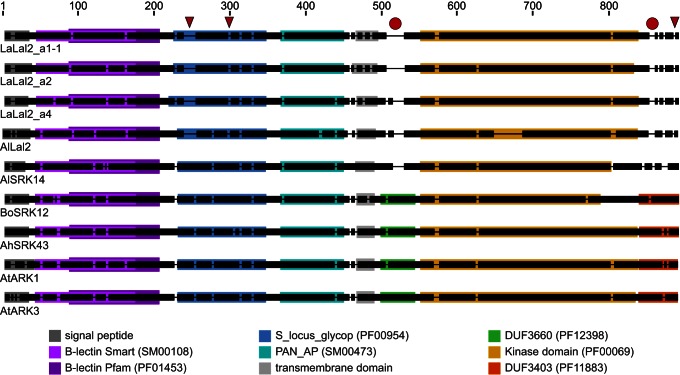
Schematic representation of aligned sequences and protein domain organization of *Lal2* alleles and closely related gene family members. The amino acid sequences of Leavenworthia a1-1, a2, and a4 *LaLal2* alleles, *Arabidopsis lyrata* AlLal2 (NCBI Gene ID 9305017), *A. lyrata* SRK14 (a class B *SRK* allele), *Brassica oleracea* SRK12, *Arabidopsis halleri* SRK43, as well as *A. thaliana* ARK3 and ARK1 were aligned along with their annotated domains. Thick black bars represent amino acid regions, and thin lines represent gaps of one or more amino acids introduced to optimize the alignment. Red arrowheads highlight alignment gaps observed specifically in all Lal2 sequences. Red circles indicate alignment gaps found in region of all Lal2 sequences and in AlSRK14 corresponding to the DUF3660 and DUF3403 domains of all other sequences. Protein domains are represented with colored boxes and their accession numbers are indicated in parentheses next to corresponding names in the color legend.

The predicted amino acid sequences of *LaLal2* and *AlLal2* have signal peptide and transmembrane domain signature sequences, as expected for a transmembrane receptor coding sequence (Figures 1 and S1B). Domain organization of LaLal2 and AlLal2 proteins predicted by the SMART/Pfam online program [31] is as follows: two overlapping B-Lectin domains, an S_locus_glycoprotein domain and a PAN_APPLE domain in their extracellular domain, and an intracellular catalytic kinase domain, the latter being made up of the 11 subdomains described for protein kinases (Figures 1 and S1B) [32].

In addition to these domains, most of the known *SRK* alleles as well as their most closely related SD-1 RLK gene family members, *ARK1* and *ARK3*, also possess DUF3660 and DUF3403 domains (Figure 1) [33]. Alignment of amino acid sequences of LaLal2 and AlLal2 to those of Brassicaceae SRK alleles (e.g., AlSRK14, BoSRK12, and AhSRK43) as well as to those of *A. thaliana* ARK1 and ARK3 produced gaps in Lal2 sequences in regions corresponding to the DUF3660 and DUF3403 domains. Although *A. lyrata* and *A. halleri* SRK sequences belonging to the class B *SRK* alleles [34] also lack these two predicted domains (e.g., AlSRK14 and AhSRK28), their sequences cluster phylogenetically within the clade of *SRK* alleles and not with the *Lal2* sequences (Figures 1, S2, and 2). Moreover, upon closer examination of the regions around the deletions of DUF3660 and DUF3403 in class B *SRK* alleles (around residues 535 and 870, respectively), the amino acid residues flanking the deletions are seen to be more similar to SRK and ARK than to Lal2 (Figure S2). There are also a number of alignment gaps that were found to be specific to all LaLal2 and AlLal2 sequences (Figures 1 and S2). Altogether, *LaLal2* and *AlLaL2* appear to be gene orthologs that code for a type of SD-1 receptor kinase that is closely related to but distinct from *SRK* sequences.

**Figure 2:**
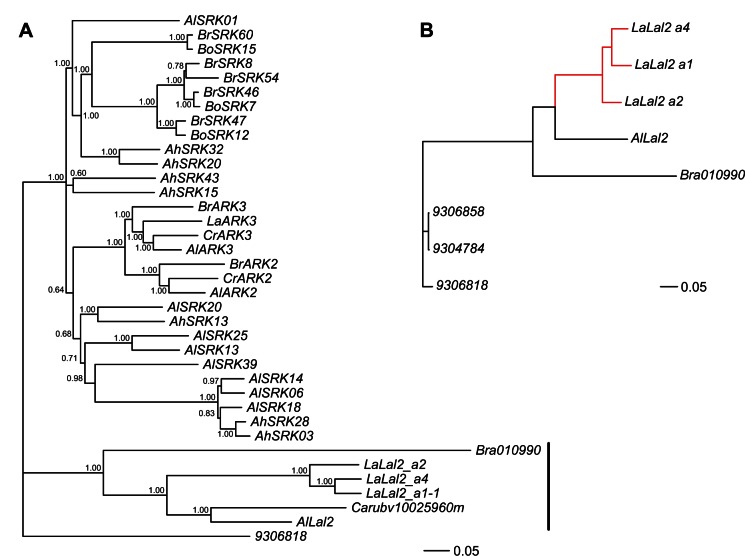
Phylogenetic reconstruction of the relationships among *Lal2*, *ARK*, and *SRK* sequences and among *Lal2*-like sequences in the Brassicaceae. Bayesian 50% consensus phylogeny for the full coding sequence of *Lal2*, *ARK*, and *SRK* sequences used in this study. (A) Posterior probabilities for each bifurcation are indicated at the nodes. *Lal2* sequences form a clade separate and distinct from *ARK* and *SRK* sequences (vertical bar). The phylogeny in (B) was generated in PhyML and used to test for codon-specific positive selection with the branch-site model. Positive selection was allowed in the foreground branches (indicated in red). Outgroups are identified by their NCBI gene ID numbers.

### Phylogenetic Analyses of the Leavenworthia *Lal2* Gene and Related Sequences

*Lal2*-like sequences were found in *Brassica rapa* (Bra010990) and *Capsella rubella* (Carubv10025960), though in genomic regions not syntenic with Leavenworthia and *A. lyrata Lal2*. Phylogenetic analysis of the full-length coding sequence of *LaLal2* alleles, *AlLaL2*, and these *Lal2*-like sequences from *C. rubella* and *B. rapa*, together with that of *SRK* and the *SRK*-related sequences (e.g., *ARK2* and *ARK3*) of other Brassicaceae species, showed that the *Lal2* group and the *SRK*-*ARK* group form two separate clades, which appear to have diverged before the onset of the strong allelic diversification of *SRK* (Figure 2A). *Lal2*-like sequences from *C. rubella* and *B. rapa* also form part of the *Lal2* clade, and show the topological relationship in the tree expected from species relationships, as do the *ARK3* sequences within the *SRK-ARK* clade [29]. Similar results were obtained when phylogenetic analysis is based only on the *S*-domain portion of the sequence, or on the transmembrane and kinase domain portions (Figure S3A and S3B), which suggests that the phylogenetic pattern of separate diversification of *Lal2* is unlikely to be due to a domain-swapping event that may have modified a hypothetical duplicate of *SRK*. Synonymous and nonsynonymous substitutions differentiating *LaLal2* and *SRK* sequences do not appear to be concentrated in any one portion of the gene (Table S2).

We applied the branch-site model test [35] to detect positive selection at individual codon sites in *LaLal2* sequences following their divergence from the most closely related sequences in the phylogeny (Figure 2B). The test rejects the null hypothesis of no selection and indicates that at least one codon (located in the hypervariable region of the *S*-domain described in [25]) has undergone positive selection (likelihood ratio test statistic = 8.426, *p*<0.005) following divergence from the other sequences.

### A Defensin-Like Encoding Gene Is Located in the Genomic Vicinity of *LaLal2*

**Figure 3:**
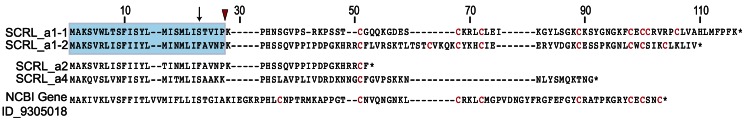
Alignment of amino acid sequences of Leavenworthia and *A.* lyrata SCRL alleles. The *A. lyrata AlSCRL* sequence corresponds to NCBI Gene ID_9305018. The a1-1 and a1-2 *LaSCRL* alleles are from the SI race and have full open reading-frames, while the a2 and a4 alleles are from SC races and encode truncated proteins. In the a1-1 and a1-2 alleles, blue box highlights the predicted signal peptide; arrow indicates conserved position of the intron; red arrowhead marks the predicted cleavage site of the a1-1 and a1-2 preproteins. Cysteines found in the predicted mature protein sequences are colored in red. Asterisks represent stop codons. Hyphens represent gaps that were introduced to optimize the alignment.

It has been noted that the *SCR* gene in previously characterized Brassicaceae *S*-locus haplotypes has the structure of a plant defensin [36]. In the three fosmid clones we sequenced, a gene exhibiting characteristics of a plant defensin was found *ca.* 2,000–10,000 bp upstream of *LaLal2*. This gene is referred to below as *SCR-like* (*SCRL*). The *LaSCRL* alleles of the a1-1 and a1-2 haplotypes contain full open reading frames and were used for further sequence analysis of the gene. Based on their cDNA sequences, we established that the *SCRL* gene consists of two exons, a characteristic common to the majority of plant defensin encoding genes [37]. Analysis with the SignalP online tool [38] predicts that the coding sequences of a1-1 and a1-2 *LaSCRL* translate into preproteins composed of an N-terminal signal peptide, required for protein secretion, and a small hydrophilic mature protein (Figure 3). The cleavage site of the signal peptide is predicted to be located after amino acid 25 in both a1-1 and a1-2 LaSCRL, generating mature proteins of 67 amino acids (aa) and 70 aa, respectively. While the signal peptide sequences of a1-1 and a1-2 LaSCRL are partially conserved (72% aa identity), the mature protein sequences are highly variable (32% identity), though like *SCR*, they contain eight cysteine residues (although their positions are not well conserved in the two sequences). Protein structure prediction using the modeling packages I-TASSER and DiANNA [39],[40] suggests that the LaSCRL product has a compact tertiary structure formed by disulfide bridges between a number of the cysteine residues, as seen in the SCRs of other Brassicaceae.

BLAST searches with the cDNA sequence or the amino acid sequence of a1-1 *LaSCRL* found only a limited number of significant hits. As with *LaLal2*, however, the genes with highest similarity are found in *A. lyrata*: genes NCBI Gene ID 9302985 and NCBI Gene ID 9305018 (Table S3), neither of which has known functions. Sequence similarity with the two *A. lyrata* genes is mainly restricted to exon 1 of *SCRL*, which corresponds to most of the signal peptide sequence. NCBI Gene ID 9302985 and NCBI Gene ID 9305018 (Figure 3) are predicted to also encode mature proteins containing eight cysteine residues and that show low sequence identity with LaSCRL. Phylogenetic analysis was not possible with *SCRL* and *SCR* sequences due to difficulties in aligning the regions.

### A Syntenic Genomic Block of *Arabidopsis lyrata* on Chromosome 7 Contains Orthologs of *LaLal2* and *LaSCRL*

**Figure 4:**
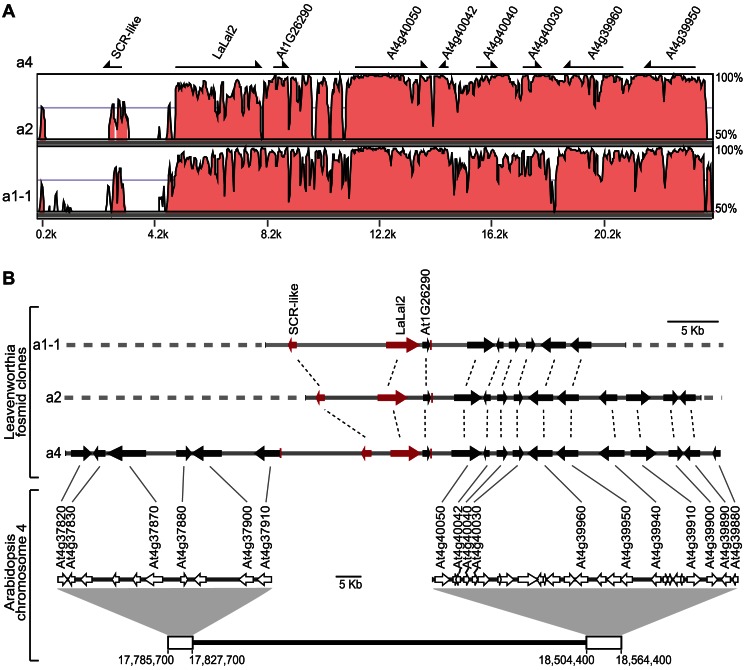
Characterization of the *S* locus genomic region in Leavenworthia. (A) VISTA alignment showing sequence conservation in a selected region of the Leavenworthia a1-1, a2, and a4 *S* haplotypes. The a4 *S* haplotype was used as the reference sequence. Arrows indicate genes annotated using the *A. thaliana* reference genome. (B) Structural gene organization of the Leavenworthia *S* haplotypes and synteny with a region of *A. thaliana* chromosome 4. Arrows represent genes in the Leavenworthia *S* haplotypes (black and red) and in the syntenic region of *A. thaliana* (white). Thick gray dashed lines represent unavailable sequences in the a2 and a1-1 *S* haplotypes. Thin dashed lines indicate orthologous genes within Leavenworthia. For clarity, only syntenic genes were identified above corresponding white arrows in the *A. thaliana* region and are connected to Leavenworthia orthologous genes by thin gray lines. Short red lines indicate the 5′ or 3′ borders of regions syntenic to *A. thaliana* chromosome 4.

Alignment of the three fosmid sequences together with sequence similarity searches in the *A. thaliana* genome database revealed that the diversity pattern in this Leavenworthia genomic region resembles the *SRK/SCR S*-locus region of other characterized Brassicaceae species [41]. The *LaLal2* and *LaSCRL* genes themselves have high sequence diversity, but are flanked (at least on the right of *LaLal2*) by highly conserved regions (Figure 4A). If we define the core *S* locus as being the region of low sequence similarity between the three haplotypes and comprising *LaLal2* and *LaSCRL*, the size of the *S* locus is 14 kb in the a4 haplotype, the only one for which sequence information on both sides of the *S* locus is available. Because the upstream sequences of the core *S* locus of the a1-1 and a2 haplotypes are currently undetermined, their sizes remain unknown, but are at least 15.3 kb in the a1-1 haplotype and 11.4 kb in the a2 haplotype. In all three Leavenworthia haplotypes, the *LaLal2* and *LaSCRL* transcription units are arranged tail-to-tail and the gene order is the same.

Annotation of the fosmid sequences using the *A. thaliana* reference genome revealed that the conserved regions on each side of the Leavenworthia core *S* locus are syntenic with an *A. thaliana* chromosome 4 region (Figure 4B). This region contains genes annotated as At4g37820 to At4g37910 on one side of the Leavenworthia core *S* locus, and genes At4g40050 to At4g39880 on the other side, but none with sequence homology to *LaLal2* or *LaSCRL*. Moreover, there are no reports of an *S* locus in this region in other Brassicaceae species that have been examined to date, including *A. lyrata*. Therefore the existence of an *S* locus in this genomic region in Leavenworthia appears to be novel.

**Figure 5:**
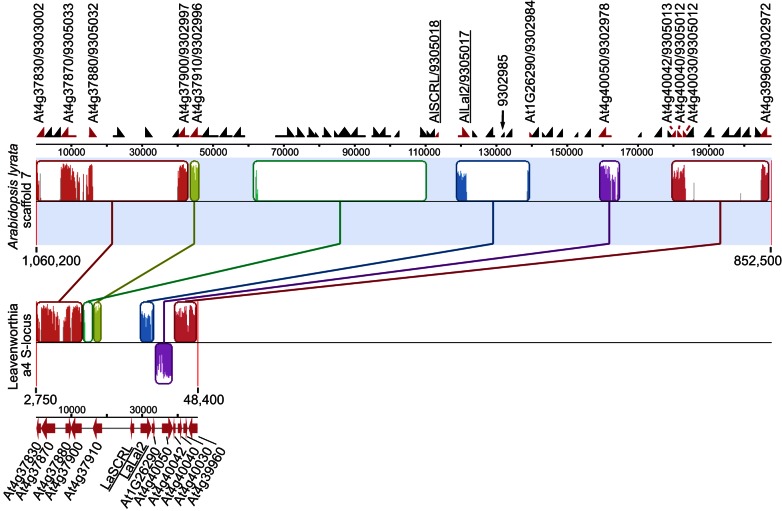
Synteny of a genomic region in *Arabidopsis lyrata* scaffold 7 and the *Lal2 S*-locus region of Leavenworthia. Mauve alignment of *A. lyrata* scaffold 7 region between positions 852,500 and 1,060,200 (from gene AT4G37830/NCBI gene ID 9303002 to AT4G39950/NCBI gene ID 9302972) and a selected region of the a4 fosmid clone sequence. Collinear and homologous regions are represented by similarly colored blocks and are connected by lines. In the Leavenworthia sequence, the purple block below the thin black line represents an inverted region. Annotated genes are shown above the *A. lyrata* panel and below the Leavenworthia panel. Genes were annotated with the *A. thaliana* reference genome, and the NCBI Gene ID numbers for *A. lyrata* genes are also given. Red arrows represent genes found in both *A. lyrata* and Leavenworthia syntenic regions; black arrows represent genes found in *A. lyrata* only. For clarity, only genes found in the syntenic region of Leavenworthia are identified, and also NCBI Gene ID 9302985. Underlined are *SCRL* and *LaLal2* genes in the Leavenworthia core *S*-locus region and their orthologous *A. lyrata* genes NCBI gene ID_9305018 (*AlSCRL*) and NCBI gene ID_9305017 (*AlLal2*).

As noted above, however, *LaLal2* and *LaSCRL* do show sequence homology to annotated but uncharacterized genes in *A. lyrata*, with highest homology to, respectively, NCBI Gene ID numbers 9305017 (called here *AlLaL2*), and NCBI Gene ID numbers 9302985 and 9305018. All three genes are located in close proximity on *A. lyrata* scaffold 7, and notably, *AlLaL2* and NCBI Gene ID 9305018 are positioned only 9.8 kb apart, and are in a tail-to-tail configuration, like *LaLal2* and *LaSCRL* in Leavenworthia (Figure 5). We refer below to the NCBI Gene ID 9305018 of *A. lyrata* as *AlSCRL*. Annotation of the surrounding genomic sequence using the *A. thaliana* reference genome revealed that this *A. lyrata* scaffold 7 region (between positions 852,500 bp and 1,060,200 bp) contains genes with annotations identical to all the genes found in the Leavenworthia a4 haplotype fosmid clone sequence. Most are homologous to genes on *A. thaliana* chromosome 4. However, a gene homologous to At1g26290 located on *A. thaliana* chromosome 1 was found in all three Leavenworthia haplotypes (between *LaLal2* and the Leavenworthia At4g40050 homolog), as well as in the *A. lyrata* syntenic genomic region (Figures 4 and 5).

In addition to the region homologous to the Leavenworthia *Lal2/SCRL S*-locus region, *A. lyrata* chromosome 7 also carries the *SRK/SCR S* locus, the latter being located at positions 9,335,860 bp (NCBI gene ID 9303924/*ARK3*) to 9,377,892 bp (NCBI gene ID 9305963/*PUB8*). The *A. thaliana* region carrying the *SRK/SCR S*-locus orthologous genes is also located between genes At4g21350 (*PUB8*) and At4g21380 (*ARK3*), in the homologous chromosome 4 region. Although the *A. lyrata* region with the homologs of the Leavenworthia *LaLal2* region genes is also on chromosome 7, it is more than 8 Mb away from the *S*-locus region.

### The Syntenic Arabidopsis *S*-Locus Region in Leavenworthia Does Not Contain *SRK* and *SCR*

**Figure 6:**
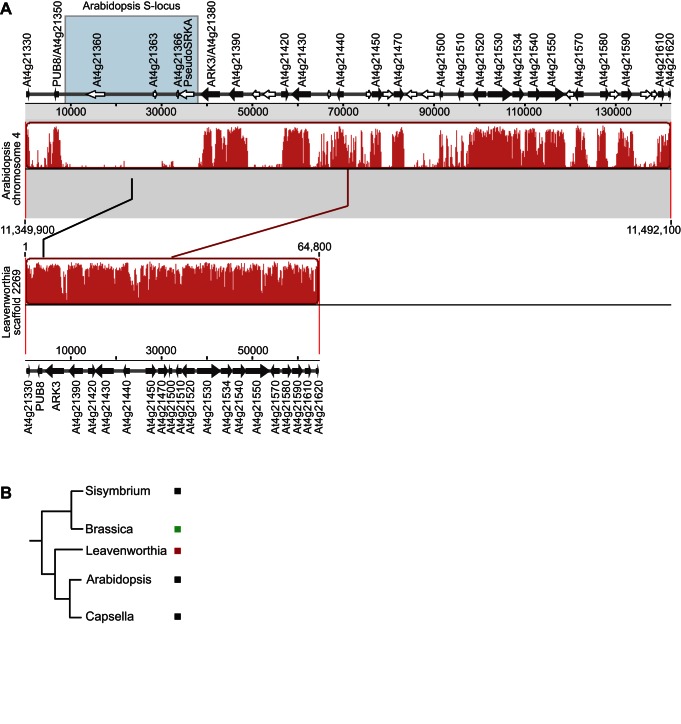
The Arabidopsis *S* locus in Leavenworthia and *S* locus positions in Brassicaceae genera. (A) Mauve alignment showing synteny of the *A. thaliana* chromosome 4 region comprised between positions 11,349,900 bp and 11,492,100 bp (from genes At4g21330 to At4g21620) and a selected region of 64,800 bp of Leavenworthia genome scaffold 2269. Annotated genes are shown above the *A. thaliana* panel and below the Leavenworthia panel. Black arrows represent genes found in both *A. thaliana* and Leavenworthia syntenic regions; white arrows represent genes found in *A. thaliana* only. Blue box highlights the *A. thaliana* core *S*-locus region that corresponds to a large deletion in Leavenworthia. For clarity, only syntenic genes and genes found in *A. thaliana* core *S* locus are identified above corresponding arrows. (B) Phylogeny of five Brassicaeae genera for which *S* locus synteny information is available. Black square denotes that the *S* locus is found in a region flanked by genes At4g21350 (*PUB8*) and At4g21380 (*ARK3*). Green square denotes that the *S* locus is found in a region flanked by genes At1g66680 and At1g66690. Red square denotes that the *S* locus is found in a region flanked by genes At4g37910 and At4g40050.

Conversely, we were able to identify the Leavenworthia genomic region carrying the homologs of the Arabidopsis *SRK/SCR S*-locus genes from data obtained in an ongoing project to sequence the *Leavenworthia alabamica* race a4 plant genome (http://biology.mcgill.ca/vegi/index.html). This Leavenworthia genomic scaffold is syntenic to genomic blocks found in the *SRK/SCR S*-locus region of *A. thaliana* (Figure 6A). Of special interest is the observation that the genomic block located between *PUB8* and *ARK3*, which contains the *SRK* and *SCR* genes in Arabidopsis species, is highly reduced in length in *L. alabamica*, which is 1.1 kb from the stop codon of the *ARK3* ortholog to the start codon of the *PUB8* ortholog (versus 4231 bp in the shortest *A. lyrata S* locus sequenced to date [41]), and neither *SRK* or *SCR* is present. PCR amplification and sequencing of the *ARK3-PUB8* region in an a1-1 *S* haplotype homozygote plant confirmed the absence of *SRK* and *SCR* orthologs in that region in a SI individual as well (Figure S4). This result is consistent with earlier crossing studies that showed that *Lal8*, the putative Leavenworthia *ARK3* ortholog, does not co-segregate with SI reactions [25]. Other *PUB8* and *ARK3* orthologs were not found in any other Leavenworthia genomic region.

It is informative to compare *S* locus locations in different Brassicaceae species for which data are available. To date, *S* loci have been reported in three different synteny blocks. As part of the genome sequencing project mentioned above, we were also able to determine that *Sisymbrium irio* has a putative *SRK* ortholog with an apparently intact open reading frame (despite the fact that this species is self-compatible), with a location similar to that of Arabidopsis *SRK* gene (Figure S5). In *Capsella rubella* [42], the *S* locus also occupies a genomic region syntenic to the Arabidopsis *SRK/SCR S* locus [on scaffold 7, between positions 7,520,515 bp (Carubv10007030m/*ARK3*) and 7,563,814 bp (Carubv10005064m/*PUB8*)]. In Brassica, the *S* locus genomic location is different, lying between orthologs of *A. thaliana* At1g66680 and At1g66690 [on chromosome 1 of *Brassica rapa*, between positions 17,225,424 bp (Bra004178/At1g66680) and 17,282,231 bp (Bra4183/At1g66690)] [43]–[45]. The *S* locus locations and phylogenetic relationships of these genera are summarized in Figure 6B, which suggests that the Arabidopsis *SRK/SCR S* locus location is ancestral.

### Expression Pattern Analysis of *Lal2* and *SCRL* in Leavenworthia and *A. lyrata*

Given the conservation of sequence and synteny described above for *LaLal2* and *LaSCRL* versus *AlLal2* and *AlSCRL*, we conducted an expression pattern study by RT-PCR of the two genes in a Leavenworthia plant homozygous for the a1-1 *S* haplotype and a *A. lyrata* SI individual in an effort to determine whether they could play a role in SI, or may have played such a role earlier in the evolutionary history of *A. lyrata*.

**Figure 7:**
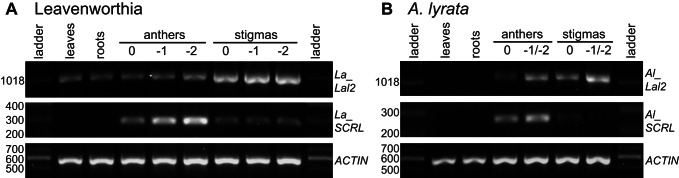
Expression pattern analysis of *Lal2* and *SCRL* by RT-PCR in vegetative and reproductive tissues. (A) Expression of the *LaLal2* and *LaSCRL* in a Leavenworthia plant homozygous at the a1-1 *S* haplotype. (B) Expression of *AlLal2* and *AlSCRL* in a self-incompatible *A. lyrata* plant.

It was shown previously that the *SRK* gene is more highly expressed in stigmas [44],[46] and that the *SCR* gene is expressed in anthers [13],[44] in Brassica and Arabidopsis, which is concordant with their respective roles in the SI mechanism. In Leavenworthia, *LaLal2* expression was detected at similar levels in leaves, roots, and anthers and at higher levels in stigmas at the different stages of flower development (Figure 7A). In *A. lyrata*, *AlLal2* expression was detected in anthers and stigmas at the different stages of flower development but not in leaves and roots (Figure 7B). As for the *SCRL* gene, its expression in Leavenworthia was detected in anthers, most strongly 2 d or 1 d before anthesis, and at lower levels in anthers at flower opening (stage 0), and in stigmas at the different stages of flower development (Figure 7A). *LaSCRL* expression could not be detected in leaves and roots. A similar expression pattern was observed for *AlSCRL* in *A. lyrata* (Figure 7B). Although the expression of *LaLal2* is not specific to stigmas and the expression of *LaSCRL* is not specific to anthers (was also found in stigmas, which was also shown for *SCR/SP11* in Brassica when using RT-PCR [43]), their expression in stigmas and in anthers, respectively, in higher levels than in other tissues is in accordance with their involvement in the SI mechanism.

To compare the relative expression levels of *AlLal2* versus *AlSRK* and *AlSCRL* versus *AlSCR* in *A. lyrata*, we also analyzed RNAseq data obtained from flower buds (stage 12) of the MN47 strain. Our analysis indicated that *AlLal2* exhibits less than 8% of the expression level compared with that of *AlSRK*, and that *AlSCRL* exhibits less than 5% of the expression level compared with that of *AlSCR* (Table S4).

### Polymorphism Analysis of *AlLal2* and *AlSCRL*

We examined whether the *A. lyrata Lal2* and *SCRL* genes exhibit a pattern of high polymorphism that would be expected if they play a role in SI. We amplified the *S*-domain of *AlLal2* and the majority of the sequence of *AlSCRL* from 10 individuals in a single SI population (Population IND) located in Indiana [47]. PCR products were visualized on SSCP gels. Banding patterns across 10 individuals were identical for both genes, suggesting monomorphism in the population (Figure S6). We sequenced the single-stranded products for each gene, and these results show the presence of only one allele at each locus. This is in contrast to the observed high levels of polymorphism exhibited in the same population where the synonymous polymorphism for genes unlinked to *SRK* is *π_s_* = 0.013 [48], suggesting that there is no evidence for a genome-wide population bottleneck in this population.

### The SC Races of *Leavenworthia alabamica* Possess Separate Mutations in the *SCR-Like* Gene

**Figure 8:**
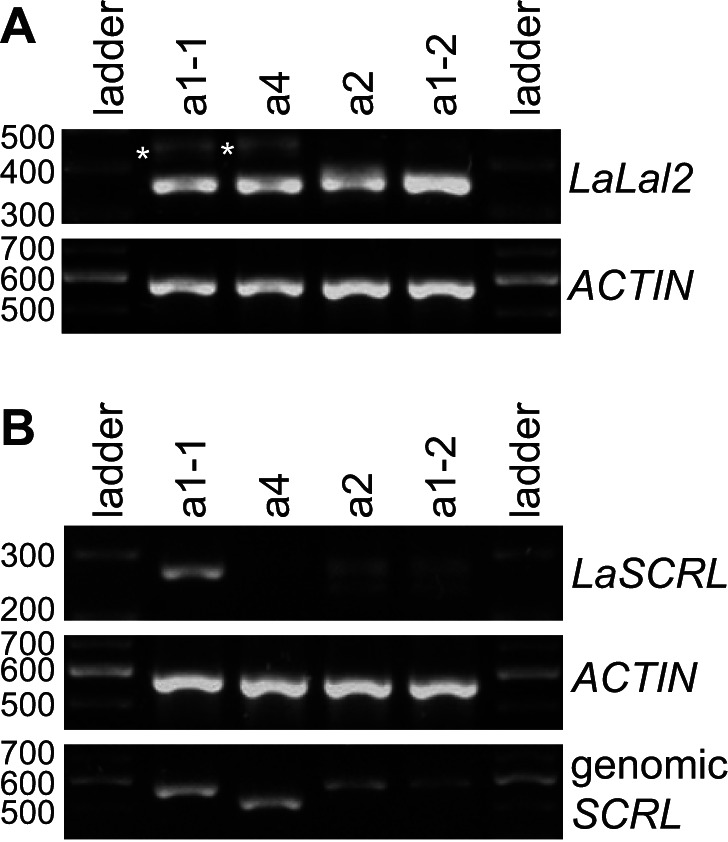
Expression analysis by RT-PCR of *LaLal2* and *LaSCRL* alleles in Leavenworthia SI and SC plants homozygous at the *S* locus. (A) Expression analysis of *LaLal2* alleles in stigmas collected 2 d before anthesis. Asterisks indicate bands corresponding to an alternatively spliced form of *LaLal2* transcripts. The *ACTIN* gene was used as an internal control. (B) Expression analysis of *LaSCRL* alleles in anthers collected 2 d before anthesis. Because of the high sequence divergence between the different *SCRL* alleles, primer pairs used for amplification were allele-specific except for the a2 and a1-2 alleles, for which the same primer pair was used. The *ACTIN* gene was used as an internal control. Genomic DNA extracted from the four haplotypes was used to amplify *SCRL* with their respective primer pairs to show that all the primer pairs used in PCR reactions amplify *SCRL*.

The sequences of the a2 and a4 *S* haplotypes were obtained with the goal of determining the nature of loss of SI in these Leavenworthia SC races, particularly by analyzing sequences and expression of *LaLal2* and *LaSCRL* in plants homozygous for the a1-1, a2, or a4 haplotypes. We included in these analyses the a1-2 haplotype found in SI plants of the a1 race. The a1-2 *LaLal2* allele encodes an *S*-domain sequence identical to that of the a2 allele (Figure S7), and these two alleles should therefore have the same SCRL pollen specificity. None of the *LaLal2* allele sequences includes any mutations disrupting the coding sequence (Figure S1B). Using stigmas of flower buds 2 d before anthesis, we found that *LaLal2* is expressed at similar levels in plants homozygous for each of the *S*-locus haplotypes described in this study (Figure 8A).

In contrast, analysis of *LaSCRL* sequences and expression revealed that the a2 and a4 alleles, from the SC races, have various disruptive mutations. In our race a4 plant, no *LaSCRL* expression could be detected in anthers 2 d before anthesis (Figure 8B), a development stage at which the a1-1 *LaSCRL* allele is highly expressed (Figure 7A). The coding region of the a4 *LaSCRL* allele deduced from the genomic DNA sequence contains a premature stop codon and the cleavage site of the signal peptide appears to be defective compared to that of the a1-1 and a1-2 *LaSCRL* alleles (Figure 3). Expression of the a2 *LaSCRL* allele was detected in anthers 2 d before anthesis (Figure 8B), but its translated sequence differs from that of a1-2 by one amino acid residue, and there is a premature stop codon after amino acid residue 45 (Figure 3). We crossed plants homozygous for the a1-2 haplotype or the a2 haplotype, to determine whether their incompatibility reactions fit those expected based on the sequence differences outlined above. The plant with the a1-2 haplotype appears to be compatible as a pollen recipient when a2 plants are used as pollen donors (89% of nine crosses produced fruit or had germinated pollen tubes). In contrast, the reciprocal crosses (a2 recipient plants and a1-2 pollen donors) appear to be incompatible with only 10% of 20 crosses that produced a fruit or had germinated pollen tubes. These proportions are significantly different (Z = 4.135, *p*<0.001) and support the hypothesis that SC in the a2 race is due to a mutation in *SCRL* (a1-2 pollen was shown to produce offspring when used in crosses with other pollen recipients). These results suggest that, as in other Brassicaceae, Leavenworthia possesses an *S* locus, which when disrupted leads to SC. Loss of SI in Leavenworthia a2 and a4 races is probably not due to loss of *LaLal2* function, but to mutations in the male function *SCRL* gene. It is not known whether putative downstream genes in the SI pathway (e.g., *ARC1*, *MLPK*) [49]–[51] are functional or not in all race a4 plants, though *ARC1* appears to be deleted in a plant obtained from one a4 race (self-compatible) population [52].

## Discussion

### The *S* Locus of Leavenworthia Is Unusual

We have characterized the Leavenworthia *S* locus in detail and have shown that it comprises two closely linked genes located in a genomic region of low sequence conservation among Leavenworthia haplotypes, as is also the case for the *SRK*/*SCR S* locus in other Brassicaceae members [41]. The two Leavenworthia *S*-locus genes, *LaLal2* and *LaSCRL*, resemble the *S*-locus genes *SRK* and *SCR* in their sequence and expression pattern, but unlike their orthologs in populations of *Arabidopsis lyrata*, they are highly polymorphic. Phylogenetic trees constructed from Leavenworthia *Lal2* alleles [25]–[28] show a pattern of long terminal branches similar to that observed at *SRK*/*SCR S* loci [53],[54].

While our previous studies indicated the existence of a functional *S* locus in the SI Leavenworthia races, the results reported here suggest that the genes comprising the Leavenworthia *Lal2/SCRL S* locus are unlike those of other Brassicaceae *S* loci that have been characterized to date. First, in Leavenworthia, *SRK* and *SCR* are absent from the syntenic block in which they occur in Arabidopsis and its close relatives, a genomic position that appears to be ancestral in the Brassicaceae. This is true in the case of the Brassica *S* locus as well, where it has been suggested that translocation of the entire *S* locus may have occurred [44]. However, the Brassica *SRK* sequences fall within the same clade as those of Arabidopsis and its relatives, despite the significantly greater phylogenetic distance between the genera as compared to Leavenworthia and Arabidopsis. By contrast, the Leavenworthia *Lal2* sequences and their sequence homologs in other Brassicaceae taxa form a distinct clade, which appears to have diverged from the *SRK-ARK* clade before allelic diversification at *SRK* that presumably occurred at the onset of the ancestral SI system of Brassicaceae. As well, the Lal2 amino acid sequences have distinct deletions compared with those of Arabidopsis and Brassica SRKs. Finally, although the *SCR-like* gene in Leavenworthia shares several features in common with *SCR*, including high sequence diversity, a coding sequence with eight cysteine residues, and a defensin-like protein predicted to form a compact tertiary structure held together by disulfide bridges, they align too poorly with those of SCRs to be orthologous. Instead, the *LaLal2* and *LaSCRL* sequences of Leavenworthia resemble SD-1 receptor kinase and defensin-like gene family members, respectively, found in a conserved syntenic block in *A. lyrata*, on the same chromosome as the *SRK/SCR S* locus but distant from it.

### The Leavenworthia *S* Locus Appears to Have Evolved Secondarily from Paralogs of *SRK* and *SCR*

Below we propose several possible explanations that could account for the distinct characteristics of the Leavenworthia *S* locus noted above. First we address the question of the time of the duplication event that gave rise to the separate *SRK* and *Lal2* lineages, and second we address the question of the time of acquisition of pollen-pistil recognition function by *Lal2/SCRL*. Regarding the first issue, focusing on the phylogenetic relationships of the *Lal2* and *SRK* sequences as shown in Figure 2, we note that these two groups of sequences form separate clades, and that the *Lal2* group belongs to a lineage that apparently diverged from the *SRK* group before *SRK* became involved in self-pollen recognition and underwent allelic diversification. The alternative hypothesis—that there was a duplication of *SRK* that gave rise directly to *Lal2* and occurred while *SRK* was already functioning in SI and thus still undergoing allelic diversification, but before the divergence of genera Arabidopsis, Capsella, Leavenworthia, and Brassica—is unlikely for the following reasons: (1) it is at odds with the structure of the gene tree and with the high level of divergence of *Lal2* from *SRK* throughout the entire *Lal2* sequence (Table S2); (2) under this hypothesis one would expect to find a gene tree with *Lal2* and *SRK* sequences interspersed at the branch tips; and (3) if *Lal2* functioned as a pollen protein-receptor this early in the evolution of SI, one would expect the level of polymorphism at *Lal2* to be high. In earlier work we showed that there is a relatively low level of polymorphism at *LaLal2* compared with *SRK*, and we found evidence of strong positive selection in hypervariable regions of the *S*-domain thought to be involved in recognition, both in our earlier studies [28] and in the PAML branch-site model analysis described above. Strong positive selection is thought to provide an indicator of recent diversification of the *S* locus, since negative-frequency-dependent selection for new *S*-allele specificities is expected to be most pronounced when *S* allele numbers are low, as expected following recent evolution of an *S* locus, or a population bottleneck [55]. Moreover, we have shown that the *A. lyrata Lal2* and *SCRL* genes do not exhibit polymorphism.

**Figure 9:**
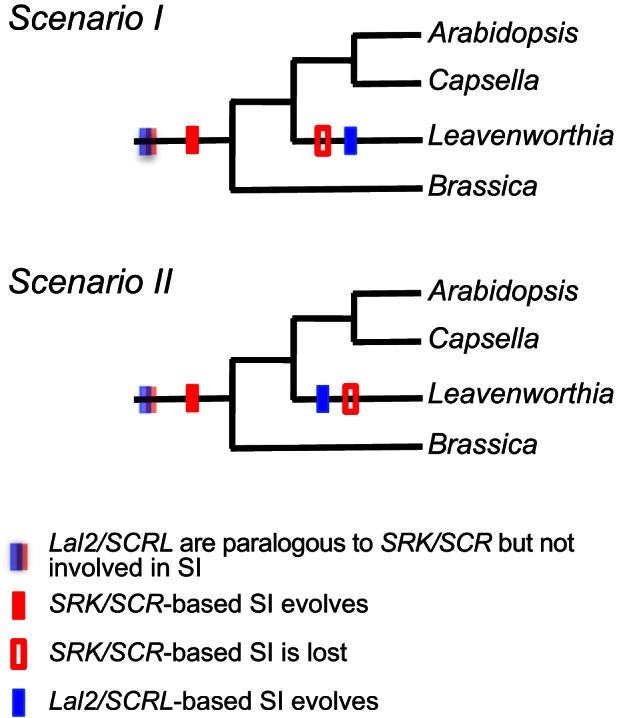
Possible evolutionary scenarios to account for the unique characteristics of the Leavenworthia *S* locus. (Scenario I) *Lal2/SCRL* pollen protein-receptor function evolves from *SRK/SCR* paralogs in the Leavenworthia lineage, following the loss of *SRK/SCR*-based SI in this lineage. (Scenario II) *Lal2/SCRL* pollen protein-receptor function evolves from *SRK/SCR* paralogs in the Leavenworthia lineage and two separate *S* loci coexist for a portion of the history of the Leavenworthia lineage, followed by eventual loss of *SRK/SCR* in this lineage.

Regarding the issue of the time of acquisition of pollen-pistil recognition function by *Lal2/SCRL*, we propose two alternative scenarios. In both cases we assume that divergence of *SRK* and *Lal2* predates the origin of SI in the Brassicaceae, and moreover, at the time of origin of SI in the family, these two genes were paralogous, with distinct functions and genomic locations. We assume that the lineage leading to *SRK* then acquired a role in SI and subsequently diversified leading to a large clade of *SRK* alleles that exhibit transgeneric polymorphism. It also likely gave rise to related genes (that do not have a function in SI) through duplication and translocation to new genomic locations unlinked to the *S* locus (e.g., *ARK1*). According to the first scenario (Scenario I), the ancestral *S* locus (i.e., with *SRK/SCR*) was lost at some point in the lineage leading to Leavenworthia, and so functional SI was lost as well (Figure 9). Pollen-pistil recognition then re-evolved based on a receptor-ligand system using the *LaLal2* and *LaSCRL* genes, with a burst of diversification. Although this scenario involves a shift in the genes involved in pollen-pistil recognition in the SI system in the Leavenworthia lineage, it is possible that the genes involved in the signaling cascade leading to inhibition of pollen germination in the incompatibility reaction have remained the same as in the other lineages. Alternatively (Scenario II) the evolution of a new *S* locus in Leavenworthia could have been a two-step process, one in which SI was never completely lost (Figure 9). This could have occurred if one gene of the new *S* locus (e.g., *LaLal2*) evolved pollen-protein recognition function, followed by evolution of a role as a protein ligand in SI for the second gene (*LaSCRL*), a series of events that could have been favored under high inbreeding depression if the ancestral system was “leaky” and allowed some selfing. Then, the original *SRK/SCR S* locus could have later been lost in Leavenworthia (perhaps following polyploidization). These two scenarios both fit the pattern of earlier divergence of *Lal2* seen in the gene phylogeny (Figure 2), and are compatible with the evidence of relatively low diversity of *Lalal2* alleles, and detection of strong selection in hypervariable regions of *LaLal2* [28].

The data from this study are insufficient to know whether SI was lost in the lineage leading to Leavenworthia (Scenario I), or whether it was retained without interruption of the SI response (Scenario II), but there are several reasons to consider that SI may have been lost in the Leavenworthia lineage before being regained. First, the loss of SI is indeed common in the flowering plants and in the Brassicaceae—it has been estimated that half the species in the family are self-compatible [56],[57], and thus, the possible loss of SI within Leavenworthia cannot be considered as an atypical event. Second, Leavenworthia has recently been shown to be a paleopolyploid species (M. Lysak, A. Haudry, M. Blanchette, personal communication). As is the case in other such taxa, the evolutionary history of Leavenworthia likely involved interspecific hybridization followed by polyploidization. Hybridization and polyploidization in an individual possessing SI may lead to loss of fertility due to the absence of mates with gametes capable of producing viable offspring, which in turn could have led to selection for the loss of SI. That is, self-fertilization (as brought about by the loss of SI) may have increased the ability of an ancestral plant to form viable offspring [58]—this is not to say that polyploidy must necessarily have led to the immediate breakdown of SI [59],[60] but rather that polyploidization could have provided a “selective filter” that favored its loss.

Clearly, Scenario I challenges the widely held notion that SI once lost is not easily regained [5],[6]. SI is, however, known to have evolved several times in the angiosperms, and so it is conceivable that it could re-evolve within the same family following loss of its pollen-pistil recognition system. It has been noted that the Brassicaceae is enriched for *S*-receptor kinase genes and these often occur near *SCR*-like genes [33]. Given the role that these genes play in recognition [7], it is possible that they could have formed the basis for the evolution of the pollen-pistil recognition system in SI in this family more than once. As well, we note that, though not specific, the expression of *Lal2* and *SCRL* in stigmas and anthers, respectively, in both *A. lyrata* and Leavenworthia suggest the presence of regulatory elements necessary to bring about a new *S* locus in the lineage leading to Leavenworthia.

It has been suggested that the loss of adaptations for outcrossing and transition to a high self-fertilization rate represent an evolutionary dead end, either because selfing lineages have higher extinction rates than outcrossing ones (due to accumulation of deleterious mutations), because of loss of adaptability, or because once lost, the purging of the genetic load leads to reduced inbreeding depression, so that outcrossing mechanisms cannot be easily regained via selection [57],[61]–[63]. If the *Lal2*/*SCRL S* locus arose following the loss of SI, the re-evolution of SI would require that the selective pressure, inbreeding depression, be retained. Theory suggests that if inbreeding depression is largely due to mutations with low selective coefficients, and if moderate levels of outcrossing persist following loss of SI, inbreeding depression may not necessarily be purged [64].

Scenario II is also interesting to consider. It would likely entail a period of evolutionary history in the Leavenworthia lineage in which two separate *S* loci could have co-existed within the same genome. SI systems with two unlinked recognition loci are known in the grasses [65].

### The Genetic Basis of SC in Leavenworthia

We found different disabling mutations at the *SCR-like* gene in different SC populations of *L. alabamica*, suggesting independent loss of SI in these populations. The same conclusion was also inferred based on phylogenetic relationships among the SI and SC populations of this species [26]. The finding that mutations in the pollen gene are involved in each case where SI has been lost in *L. alabamica* parallels recent reports in *Arabidopsis thaliana* and *A. kamchatica* [60],[66] and also lends support to a prediction from population genetic theory that mutations disabling the pollen gene (as opposed to those disabling the stigma gene) should more easily spread in populations [67]. Moreover, the loss of SI in *L. alabamica* was probably recent, as *LaLal2* genes in the SC populations are apparently still intact and expressed, and at least one of the SC *L. alabamica* populations studied here (the a2 race population) exhibits mixed selfing and outcrossing. Had the loss of SI and breakdown of *SCR*-like genes in these populations occurred in the more distant evolutionary past, it would presumably have rendered the *LaLal2* gene selectively neutral and subject to mutational decay, and we would have expected to find a signature of such decay or neutrality in *LaLal2* sequences. However, we cannot rule out the possibility that this gene also serves an additional unknown function, as suggested by the expression of *LaLal2* in tissues other than stigmas. For example, a dual function has been found for an *SRK* gene in Arabidopsis [68].

### Conclusions and Future Research

The results of this investigation suggest that *S* locus evolution in Brassicaceae is more complex than initially thought. The vast majority of molecular-level studies of SI have been conducted with a limited number of model plant systems or their close relatives [4]. The work we present here, on a non-model organism, underscores the importance of looking outside these systems to understand more broadly the evolution of SI. It will be important to examine the genetic basis of SI in more distantly related Brassicaceae species to determine whether there are other taxa with SI systems that appear not to be based on *SRK* and *SCR*.

Apart from the evidence that we have presented and discussed above, there are other types of information that could be useful in determining with greater certainty whether the *S* locus in Leavenworthia could have evolved as a duplication of the *SRK*/*SCR S* locus, rather than as a result of neo-functionalization, as we have proposed here. One potentially useful piece of information pertains to the role of *Lal2* and *SCRL* in other Brassicaceae taxa. Even though apparent *Lal2* sequence orthologs exist in other Brassicaceae species, there is no information available to test whether pollen recognition in SI is based on *Lal2* alleles in any of these taxa (apart from what we have presented for *Arabidopsis lyrata*, suggesting that it is not). To further rule out the possibility that *Lal2*/*SCRL*-based SI exists in other Brassicaceae genera, it would be valuable to explore the levels of polymorphism of *Lal2* and *SCRL* orthologs in other taxa and determine whether they are characteristic of an *S* locus. In addition, crosses could be conducted to reveal whether these genes co-segregate with SI reactions, as has been done in earlier studies that focused on the role of *SRK* and *SCR* in SI. The existence of a few rare *S* allele sequences in Leavenworthia with some similarity to those of *A. lyrata* and *A. halleri SRK*s (as noted above) could be interpreted as support for the duplication (as opposed to neo-functionalization) hypothesis. But such evidence is premature. These sequences could simply be pseudogenes that are linked to the Leavenworthia *S* locus, and therefore show cosegregation with SI. It would be useful to determine the genomic location of these sequences in the few plants where they occur, and whether they play any active role in SI.

Finally, in future research directed at understanding the evolution of the Leavenworthia *S* locus, it would also be interesting to attempt transformation of SC species of Arabidopsis or Brassica with Leavenworthia *SCRL* and *LaLal2* genes from the same haplotype, to determine whether these genes function within the context of the same downstream signaling pathway(s) as *SRK*/*SCR* [49]–[51].

## Materials and Methods

### Plant Material and Growth Conditions

*Leavenworthia alabamica* seed was sown in a 1∶1 mixture of PRO-MIX BX (Quebec, Canada) and sand. Plants used for expression analyses, genome sequencing, and fosmid cloning were grown in a Conviron PGW36 growth chamber under 14-h days at 22°C with a nighttime temperature of 18°C. Plants used for crossing were grown in a greenhouse at a minimum daytime temperature of 20°C and 18°C at night. Supplemental lighting was provided as needed to achieve a minimum day length of 12 h.

When generating plants for expression analyses and crossing, plants homozygous for functional *S*-locus haplotypes (a1-1 and a1-2) were generated through self-pollination using a saline treatment modified from [69]. The stigma of the plant to be selfed was hydrated with 0.5 M NaCl. After 1 h the stigma was then pollinated with self-pollen, either from an anther from the same flower or from another open flower of the same plant. The resulting progeny were screened for homozygosity for the allele of interest. Plants from the a2 and a4 races of *L. alabamica* are homozygous for the a2 and a4 *LaLal2 S* haplotypes, respectively. Crosses and pollen tube staining were conducted according to previously published methods [25]. Pollinations were considered compatible when more than five pollen tubes were visible in the style of the maternal parent or >1 seed was produced in the mature silique.

The *Arabidopsis lyrata* plant used for *AlLal2* and *AlSCRL* expression analysis was obtained from a seed collected in [70] and was grown in a Conviron PGW36 growth chamber under 16-h days at 22°C with a nighttime temperature of 18°C.

### Nuclei Purification and DNA Extraction

Genomic DNA samples of plants of the a1-1, a2, or a4 *S* haplotypes used in fosmid library construction were extracted from purified nuclei. Nuclei were purified from fresh or frozen plant tissues. Tissues were grinded in liquid nitrogen using a mortar and pestle. Powdered tissues were added to freshly made and ice-cold nuclei extraction buffer [10 mM Tris HCl (pH 9.5); 10 mM EDTA (pH 8.0); 100 mM KCl; 500 mM sucrose; 4 mM spermidine; 1 mM spermine; 0.1% β-mercaptoethanol] in a ratio of 20 ml of buffer per gram of tissue. Solution with added tissue was stirred using a magnetic stir bar for 10 min and then filtered through two layers of cheesecloth combined to one layer of Miracloth into a clean beaker. Cold lysis buffer (nuclei extraction buffer with 10% Triton X-100) was added at a ratio of 2 ml per 20 ml of nuclei extraction buffer. Solution was stirred for 2 min, poured into cold 50 ml polyethylene tubes, and centrifuged at 2,000 g for 10 min at 4°C to pellet nuclei. Supernatant was poured off, and the remaining supernatant was removed with a micropipette after a quick-spin.

DNA was extracted from purified nuclei using Genomic-tips 20/G and the Genomic DNA Buffer Set (Qiagen). Instructions given in the Qiagen Genomic DNA Handbook (August 2001) for Yeast starting at p. 37, step 8 were used except for this following modification: at step 9, Proteinase K was added and incubation was carried overnight with gentle shaking at 50 rpm on a MixMate Plate and Tube Mixer (Eppendorf) to lyse the nuclei. Genomic DNA samples used in standard DNA analysis were extracted with the DNeasy Plant Mini Kit (Qiagen).

### Fosmid Library Construction and Screening

Fosmid libraries were constructed using the CopyControl HTP Fosmid Library Production Kit (Epicentre Biotechnologies) as specified by the manufacturer's instructions with the following modifications and specifications. Genomic DNA was sheared by passing gDNA samples 35 times through a Gastight 10 µl Hamilton syringe (model 1701). Sheared DNA was end-repaired and submitted to size separation by migration in a 1% low melting point agarose gel for 36 h at 35 V in 0.5× TBE buffer. Insert DNA ranging from 23 to 40 kb was recovered from the gel matrix using GELase. We used 250 µg of purified DNA for ligation into the pCC2FOS Vector. After titering the packaged fosmid clones, cells were grown overnight at 37°C in liquid gel pools [71],[72] in 96-deep-well plates at a density of either 100 or 250 cfu per pool [200 µl of LB SeaPrep Agarose (Lonza Rockland Inc.) supplemented with 12.5 µg/ml chloramphenicol (Cam)].

Clones containing the *Lalal2* gene were isolated by doing successive rounds of PCR screening on library pools of decreasing number of clones. In the first round, an aliquot of several library pools were combined to create superpools. Cells were pelleted by centrifugation and resuspended in sterile water. An aliquot of 0.5 µl each of resuspended cells was used in standard PCR reactions. In the second round, pools from the obtained positive superpools were screened. In the third round, positive pools were plated on LB agar plates supplemented with 12.5 µg/ml Cam to get isolated colonies. Colonies were individually picked and combined into pools of 10 colonies for PCR screening. Final screening round was carried on individual colonies grown on LB agar+12.5 µg/ml Cam plates from positive pools of 10.

To increase sensitivity of the screening, each round of screening consisted of two successive rounds of PCR reaction (primary and secondary). Primary PCR reactions were carried with primer pair Lal-Sdomain5′-F and Lal-Sdomain3′-R. Secondary PCR reaction used nested primer pair LalGenF and LalRcon. See Table S5 for primer sequences.

### RNA Extraction and Expression Analysis

Total RNA samples were extracted from plant tissues by using the RNeasy Plant Mini Kit (Qiagen). RNA samples were purified from DNA contamination by carrying an on-column treatment with DNase as specified in the manufacturer's instruction manual. For expression analysis of *Lal2* and *SCRL* by RT-PCR, 1 ug of total RNA was used in reverse transcription reactions using SuperScript II Reverse Transcriptase (Invitrogen, Burlington, ON) and Oligo(dT)_12–18_. The 5′/3′ RACE reactions were carried with the FirstChoice RLM-RACE Kit (Invitrogen) using 2 ug of total RNA. The 5′ adapter-ligated RNA was reverse transcribed with the M-MLV Reverse transcriptase provided with the kit and using either random decamers or the 3′ RACE adapter as primers. PCR amplifications on reverse-transcribed products were carried using the following conditions: 1 µl RT products, 1× PCR buffer, 0.2 mM dNTP mix, 2 mM MgCl_2_, 0.4 µM forward primer, 0.4 µM reverse primer, and 0.75 U Taq Polymerase (Invitrogen), in a final volume of 20 µl. PCR cycling was done in a C1000 thermal cycler (Bio-Rad) using the following program: initial denaturation at 94°C, 5 min followed by 35 cycles at 94°C, 30 s; 58°C, 30 s.; 72°C, 1 min; and a final elongation step at 72°C, 5 min. See Table S5 for primer sequences.

Illumina RNAseq reads from *A. lyrata* seedlings, roots, and stage 12 flowerbuds obtained courtesy of Dr. Richard Clark and Joshua Steffen were obtained using methods described in [73]. RNAseq reads were aligned to the *A. lyrata* reference genome (strain MN47: JGI) using both novoalign (Novocraft) and spliceMap (PMID: 20371516). Novoalign was used in read quality re-calibration mode with a low level of mismatch permitted (t = 50) between read and reference. Independently spliceMap was used to map reads spanning exon junctions. For each gene model, an expression level was determined by adjusting the read-count per gene by the exon-length and total reads in the respective sequencing libraries.

### DNA Sequencing and Sequence Analysis

Sanger, Illumina, and 454 sequencing were performed at the McGill University and Genome Quebec Innovation Centre. The genomes of *Leavenworthia alabamica* (a4 race), *Sisymbrium irio*, and the *Leavenworthia* short read data were gathered as part of an ongoing comparative genomics investigation involving these and other Brassicaceae species (Blanchette et al., unpublished data). The sequences of the a1-1, a2, and a4 fosmid clones were also assembled from 454 data. In the case of the genomes, reads were generated in accordance with the Illumina protocols, with special attention paid to gentle shearing of mate-pair circular DNA to ensure >500 nt fragments, thereby reducing the probability of a read fragment-join chimera. Paired end (2×105, nominal 64 nt gap) Illumina reads were generated to a depth of 80× for each genome, trimmed for quality (3′ trimming where Q<32) and assembled with the Ray assembler [74] using automatic coverage depth profiling and a Kmer of 31. Scaffolding of Ray contigs was then undertaken with the SOAPdeNovo (BGI) assembler using a combination of 5 and 10 KBase mate pair reads (Blanchette et al., unpublished data). Assembly of the fosmid sequences was undertaken in batches of pooled barcoded libraries covered by 1/8 of a flowcell of 454 sequencing (200× coverage). After stripping vector contaminants Newbler (Roche) was used to assemble the reads into ∼40 Kbase contigs using essentially default assembly parameters. Comparison of targeted fosmid assemblies (454) and short read whole genome assemblies (Illumina-Ray) from *L. alabamica* of the a4 race demonstrated high levels of concordance.

Standard sequence analyses were done using the Geneious v. 5.4.6 software (Auckland, New Zealand) [75]. Amino acid and nucleotide sequences were aligned with MUSCLE [76]. Fosmid sequences were aligned using VISTA [77]. Annotation of fosmid sequences was done by sequence blast against the *Arabidopsis thaliana* genome. Because of the high sequence diversity of *LaSCRL*, this gene could not be detected by blast search but was found by eye examination of short ORFs obtained from different translation frames for the presence of eight cysteines. The Mauve Genome Alignment software v. 2.2.0 [78] was used to compare the *S* locus of *A. thaliana* with syntenic genome region of Leavenworthia and the *S* locus of Leavenworthia with syntenic genome region of *A. lyrata*. Protein domains were determined by submitting gene amino acid sequences to the SMART/Pfam prediction tools [31].

### Phylogenetic Analyses

In addition to the a1-1, a2, and a4 *LaLal2* sequences, we selected full-length coding *SRK*, and the closely related receptor-like kinase genes *ARK1*, *ARK2*, and *ARK3* sequences from several Brassicaceae taxa. We included the coding sequence of *AlLal2* (NCBI gene ID 9305017), the *A. lyrata* gene showing apparent orthology to *LaLal2* as based on sequence similarity and conserved synteny (see above). Sequences homologous to *Lal2* were identified in *Capsella rubella* (Carubv10025960m) and *Brassica rapa* (Bra010990). This was done as follows. First, pairwise alignments were generated between *A. lyrata* and *L. alabamica*, *C. rubella*, and *Brassica rapa* genomes, using lastz [79] in gapped, gfextend mode. These alignments were then chained [80] to generate extended sets of alignments split by gaps of less than 100 KBase. Low scoring chains were rejected and a subset of the highest scoring chains were annotated as candidate orthologous alignments between pairs of genomes. For the *L. alabamica* and *B. rapa* genomes, up to three orthologous chains were permitted for each region of the *A. lyrata* genome to represent orthology between the diploid and hexaploid contexts. The remaining chains were annotated as candidate homologous alignments. These alignment chains were used to identify candidate orthologs and homologs. The *AlLal2* (NCBI gene ID 9305017), Carubv10025960, and Bra010990 predicted coding sequences were edited by sequence alignment of their genomic sequences with the Leavenworthia and *A. lyrata Lal2* cDNA sequences obtained by sequencing. The outgroup for the analysis was selected from the sequences on the basis of closeness in evolutionary distance to the ingroup sequences as suggested by [81], from the Brassicaceae family RLK sequences examined in [33].

The sequences were aligned using the default settings in Clustal Omega v. 1.1.0 [82], and the best-fit nucleotide substitution model for the alignment was determined by the Aikake Information Criterion as implemented in jModeltest v.0.1.1 [83],[84]. MrBayes v. 3.1.2 [85] was used to carry out Bayesian phylogenetic inference under the GTR+I+Γ substitution model. All parameters were estimated during two independent runs of six Markov Monte Carlo chains, both of which were run for 4,000,000 generations (longer runs gave identical results). Phylogenetic trees were sampled every 4,000^th^ generation, and a consensus phylogeny was built from the 751 trees remaining after the first 250 were discarded as burn-in. Nexus formatted alignments including the commands used in MrBayes are available from the Dryad Digital Repository: http//dx.doi.org/10.5061/dryad.mq5ct [86].

The branch-site model test for positive selection at codon sites was carried out using the CODEML program in the PAML 4.4 package [87]. The tree (Figure 2B) was obtained using the PHYML [83] with default settings as implemented in Geneious v. 5.4.6 [75]. Foreground branches for the branch-site model were assumed to be those in which *LaLal2* evolved separately from related sequences in Figure 2B.

### Analysis of Synonymous and Nonsynonymous Substitution

To determine whether sequence evolution of *Lal2* associated with *S* locus evolution in this group was concentrated into particular protein domains, we compared the sequence of the a1-1 haplotype with that of the phylogenetically closest *SRK* sequence (allele *SRK15* from *Arabidopsis halleri*). Estimates of synonymous and nonsynonymous substitution and their ratios were obtained by maximum likelihood using the program CODEML in the PAML package [87]. Estimated parameters for each major protein domain were compared by constraining them to be equal and carrying out the log likelihood ratio test.

### Polymorphism Analysis of *AlLal2* and *AlSCR*

We amplified portions of *AlLal2* and *AlSCR* from 10 individuals from the IND population of *A. lyrata* (material obtained courtesy of Dr. Barbara Mable) [47]. Polymorphism data of genes unlinked to the *S* locus were obtained from [48]. PCR primers are reported in Table S5, and PCR reaction protocols were identical to those reported above for RT-PCR. Amplicons were run on single-strand conformational polymorphism (SSCP) gels, as described in [28],[88]. Bands corresponding to single-stranded products of *AlLal2* and *AlSCRL* were cut from the gel, re-amplified, and sent for Sanger sequencing at the McGill University and Génome Québec Innovation Centre (Montreal, Canada). Sequence trace files were edited by eye in Geneious v. 5.4.6 [75] and aligned to the reference copies of *AlLal2* (100% identity) and *AlSCRL* (99.8% identity).

### Sequence Data

Sequences unique to this study were deposited in GenBank.

## Supporting Information

Figure S1 Sequence analysis of *LaLal2*. (A) Schematic representation of the alignment of the a4 *LaLal2* genomic DNA and cDNA sequences. Exons are represented with white boxes and their sizes in bp are indicated in parentheses. (B) Alignment of predicted amino acid sequences of the a1-1, a2, and a4 alleles of LaLal2. Amino acid sequences were deduced from cDNA sequences. Consensus sequence is shown above allele sequences, with X representing residues not conserved in the three alleles. Sequences of the predicted protein domains determined by the SMART/Pfam programs for the a1-1 *LaLal2* allele are highlighted using the color code shown below. Red arrowheads indicate the 12 conserved cysteine residues in the extracellular domain. The kinase domain possesses the 11 kinase subdomains (I to XI) as established by [32].
(PDF)

Figure S2 Amino acid sequence alignment of *Lal2* alleles and closely related sequences. Leavenworthia *LaLal2* alleles, *A. lyrata* AlLal2 (NCBI Gene ID 9305017), Lal2-like sequences from *B. rapa* (Bra010990) and *C. rubella* (Carubv10025960m), a selection of full-length coding sequences of *SRK* alleles from *A. lyrata*, *A. halleri*, and *Brassica sp.*, as well as *A. thaliana* ARK3 and ARK1 were aligned. AlSRK14 and AhSRK28 belong to class B *SRK* alleles. Consensus sequence is shown above sequences, with X representing residues not conserved. The approximate positions of protein domains are indicated below the aligned sequences. Dashes represent gaps introduced to optimize the alignment. Red arrowheads highlight alignment gaps observed specifically in all Lal2 sequences. Red circles indicate alignment gaps found in the regions of all Lal2 sequences and in class B *AlSRK14* and *AhSRK28* alleles corresponding to the DUF3660 and DUF3403 domains in all other sequences.
(PDF)

Figure S3 Phylogenetic reconstruction of the relationships among *Lal2*, *Lal2*-like, *ARK*, and *SRK* for different portions of the sequence. Bayesian 50% consensus phylogeny for the S-domain (A) and the transmembrane and kinase domains (B) of *Lal2*, *Lal2*-like, *ARK*, and *SRK* sequences used in this study. Posterior probabilities for each bifurcation are indicated at the nodes. *Lal2* sequences form a clade separate and distinct from *ARK* and *SRK* sequences (vertical bars). The outgroup in each tree is identified by its NCBI gene ID number.
(PDF)

Figure S4 Sequence alignment of the *ARK3*-*PUB8* intergenic region in Leavenworthia SC a4 and SI a1-1 plants. Highlighted in blue are the 3′ end of the coding sequence of *ARK3* (top) and the 5′ end of the *PUB8* (bottom) orthologs. The a4 sequence was extracted from Leavenworthia scaffold 2269 (Figure 6A). The a1-1 sequences were obtained by PCR amplification using primers anchored in the *ARK3* and *PUB8* coding sequences, followed by end-sequencing of PCR products (size of about 1.5 kb). Note that the a1-1 end sequences obtained do not overlap and the sequence corresponding to a stretch of 45 nt of the a4 sequence (between positions 650 and 696) remains unknown. Green horizontal bars above aligned sequences indicate identity between sequences. The *ARK3*-*PUB8* intergenic regions covered by the a1-1 sequences are 93% identical between a1-1 and a4.
(PDF)

Figure S5 Genomic organization of the *S* locus in *Sisymbrium irio*. An *SRK* gene sequence was identified in a genome region between gene orthologs of *A. thaliana PUB8* and *ARK3*. Genes were annotated using the *A. thaliana* reference genome.
(PDF)

Figure S6 SSCP gel for *AlLal2* and *AlSCRL* from 10 individuals from a single *A. lyrata* population. The observed banding patterns indicate monomorphism for both loci (see text for details).
(PDF)

Figure S7 Alignment of the a2 full-length and a1-2 partial LaLal2 amino acid sequences. The a1-2 aa sequence was deduced from cDNA sequence obtained by using primers anchored in exon 1 and exon 7 of the gene (see Table S5 for primer sequences) and corresponds to positions 169 to 714 of the a2 LaLal2 aa sequence. Green horizontal bars above aligned sequences represent identity between sequences. Note that the available aa sequence of a1-2 is identical to that of a2 except for one amino acid residue located in the intracellular kinase domain. The predicted transmembrane domain is highlighted with a blue box to delimit the extracellular domain versus the intracellular domain.
(PDF)

Table S1 Highest matches obtained in BLASTn searches using the full-length genomic sequence of the Leavenworthia a1-1 *LaLal2* allele.
(XLSX)

Table S2 Estimates of the ratio and rates of nonsynonymous and synonymous substitution per site for four major protein domains in a comparison of *Lal2* and *SRK* coding sequences.
(DOCX)

Table S3 Highest matches obtained in BLASTn searches using the cDNA (A) or the amino acid (B) sequences of the a1-1 *LaSCRL* allele.
(XLSX)

Table S4 RNAseq expression analysis of *AlLal2*, *AlSCRL*, *SRK*, and *SCR* in *Arabidopsis lyrata* strain MN47.
(DOCX)

Table S5 List of PCR primers used.
(XLSX)

## Abbreviations

SI: self-incompatibility
SC: self-compatibility
*SCR*: *S*-locus cysteine-rich gene
*SRK*: *S*-locus receptor kinase
