# Supplementary material for: Secondary Evolution of a Self-Incompatibility Locus in the Brassicaceae Genus Leavenworthia
Source: PLoS Biol. 2013 May 14;11(5):e1001560. doi: 10.1371/journal.pbio.1001560 (PMC3653793; doi:10.1371/journal.pbio.1001560)
Supplement: Figure S1 — Sequence analysis of LaLal2. (A) Schematic representation of the alignment of the a4 LaLal2 genomic DNA and cDNA sequences. Exons are represented with white boxes and their sizes in bp are indicated in parentheses. (B) Alignment of predicted amino acid sequences of the a1-1, a2, and a4 alleles of LaLal2. Amino acid sequences were deduced from cDNA sequences. Consensus sequence is shown above allele sequences, with X representing residues not conserved in the three alleles. Sequences of the predicted protein domains determined by the SMART/Pfam programs for the a1-1 LaLal2 allele are highlighted using the color code shown below. Red arrowheads indicate the 12 conserved cysteine residues in the extracellular domain. The kinase domain possesses the 11 kinase subdomains (I to XI) as established by [32]. (PDF) [file pbio.1001560.s001.pdf]

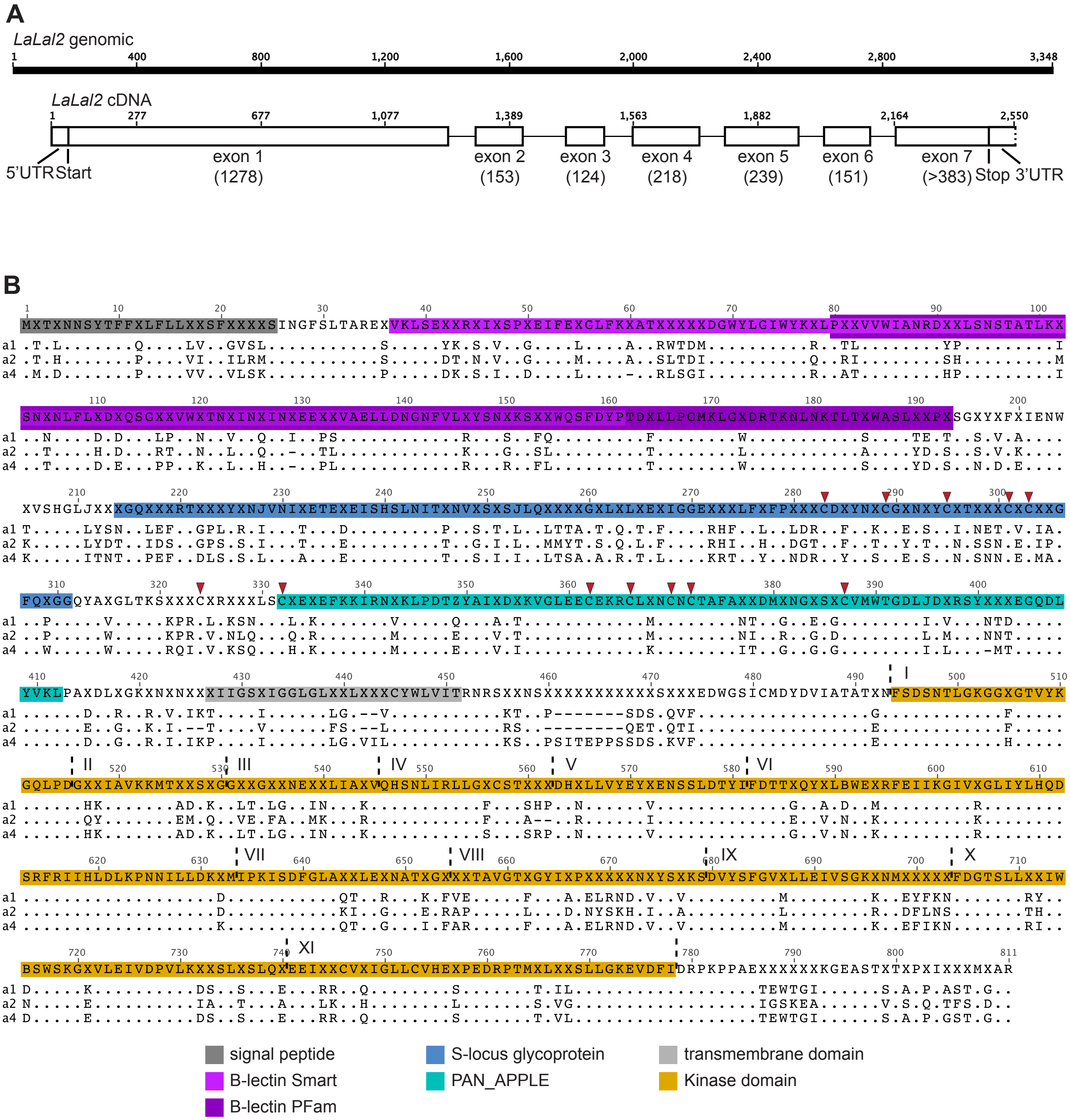
A
LaLal2 genomic
1
400
800
1,200
1,600
2,000
2,400
2,800
3,348
LaLal2 cDNA
1
277
677
1,077
1,389
1,563
1,882
2,164
2,550
5'UTR
Start
exon 1 (1278)
exon 2 (153)
exon 3 (124)
exon 4 (218)
exon 5 (239)
exon 6 (151)
exon 7 (>383)
Stop
3'UTR
B
signal peptide
B-lectin Smart
B-lectin PFam
S-locus glycoprotein
PAN_APPLE
transmembrane domain
Kinase domain
