# Supplementary material for: Secondary Evolution of a Self-Incompatibility Locus in the Brassicaceae Genus Leavenworthia
Source: PLoS Biol. 2013 May 14;11(5):e1001560. doi: 10.1371/journal.pbio.1001560 (PMC3653793; doi:10.1371/journal.pbio.1001560)
Supplement: Figure S2 — Amino acid sequence alignment of Lal2 alleles and closely related sequences. Leavenworthia LaLal2 alleles, A. lyrata AlLal2 (NCBI Gene ID 9305017), Lal2-like sequences from B. rapa (Bra010990) and C. rubella (Carubv10025960m), a selection of full-length coding sequences of SRK alleles from A. lyrata, A. halleri, and Brassica sp., as well as A. thaliana ARK3 and ARK1 were aligned. AlSRK14 and AhSRK28 belong to class B SRK alleles. Consensus sequence is shown above sequences, with X representing residues not conserved. The approximate positions of protein domains are indicated below the aligned sequences. Dashes represent gaps introduced to optimize the alignment. Red arrowheads highlight alignment gaps observed specifically in all Lal2 sequences. Red circles indicate alignment gaps found in the regions of all Lal2 sequences and in class B AlSRK14 and AhSRK28 alleles corresponding to the DUF3660 and DUF3403 domains in all other sequences. (PDF) [file pbio.1001560.s002.pdf]

```
                   1        10        20        30        40        50        60        70        80
Consensus          MXXXXXXXXXXXXXXXXXXXXXXXXXXXXXXXXXXXXXXXXXXXXXXXJXSXXXXFEXGXFXXXXXXXXXXXXWYLGXWX
1. La_Lal2_a1-1    .TT-LNNSY------TFFQLFLLLVSFGVSLSINGFSLTARESVKLSEYKRSIV.PGEI..L.L.KAATRWTDMDG....I.Y
2. La_Lal2_a2      .TT-HNNSY------TFFPLFLLVISFILRMSINGFSLTARESVKLSEDTRNIV.PGEI..M.L.KAATSLTDIDG....I.Y
3. La_Lal2_a4      .MT-DNNSY------TFFPLFLLVVSFVLSKSINGFSLTAREPVKLSEDKRSII.PDEI..L.L.K-ATRLSGIDG....I.Y
4. Al_Lal2         .TMTRSVPHGNHFYTSFFFFVFQLVVLIPSIASYDSTFSPTRPLRITEN-ETIV.PEGI..L.F.KPATRFQERDR....I.Y
5. Carubv10025960m                                             MNLGEDKGTIV.PGKI..L.L.KGTTSVPYIDR....I.Y
6. Bra010990                   MFRRLSFLVFIFTSVLSFEVW---------FSEN-ERIV.PSSI..L.L.KDRT------G....I.F
7. Al_SRK14        .RGVIPKYH--QSHNFFFFVFVVSTLFLPALSIYANTLLSTESLTIASN-QTIV.LGDD..L.F.KPAASLRDGDR....I.Y
8. Ah_SRK28        .RGAVPNYH--HFHNFFFFLFVVSVLFCPAFSIFANTLSSTESLTIASN-QTIV.LGDD..L.F.RPAASLREGDR....I.Y
9. Al_SRK01        .RG-VRSIYHHSITLCFFAVLVVLILFCCAFSIHANTLSSTESLTISRN-LTIV.PGKI..L.F.KPSTRP----R....I.Y
10. Al_SRK25       .RGEVPNKH---HSYTFFFVLFFALVLFPDFSISANTLSATESLTISSN-KTIV.PGGV..L.F.KIL-----GDS....I.Y
11. Ah_SRK13       .RA-LPNNH-------HFYILVIFFLLRSALPINVNTLSSTESLTISSN-RTIV.LGDV..L.F.NPTPSSRDGDR....I.Y
12. Ah_SRK43       .RG-FRNIYRHSHTFSFLLVFFVLILFFPAFSSTVNTLSATESLTISSN-RTIV.PNDV..L.F.KPGTSS----R....I.Y
13. Bo_SRK12       .KG-VRNIY-HHSYSSFLLVFVVTILFHPALSIYINTLSSTESLTISSN-RTLV.PGDV..L.F.ETNS------R....M.Y
14. Br_SRK54       .KG-VQNSY----TFCFLLVFVVLILVHPALSIYFNILSSTESLTISGN-RTLV.PGDV..L.F.RTTSSS----R....I.Y
15. Br_SRK60       .KG-VHNIYHHSYTFSFLLVFLALILFHPALSTYVNTMSSSESLTISSN-RTLV.PGGV..L.F.KPSGRS----R....I.Y
16. At_ARK1        .RS-VPNYH------HSFFIFLILILFL-AFSVSPNTLSATESLTISSN-KTII.PSQI..L.F.NPASSS----R....I.Y
17. At_ARK3        .RG-LPNFY-----HSYTFFFFFLLILFPAYSISANTLSASESLTISSN-NTIV.PGNV..L.F.KPGLDS----R....I.Y
                   [signal peptide]                                        [B-lectin (SM00108)]

                         90        100       110       120       130       140       150       160
Consensus          XXXXXXXXXWXXNRXXXJXXXXXXLXXXXXXXJXXXXXXXXXXXWXXXXXXXXXXXXXXXXXXLXXXGNXXXXXXXXXXXXX
1. La_Lal2_a1-1    KRLPTLV-V.IA..DYPLSNSTAT.KISNNN--LFLDDDQSGLPV.NTNVINQ-INIEEPSVAE.LDN..FVLRYSNSK---S
2. La_Lal2_a2      KQLP-RIVV.IA..DSHLSNSTAT.KMSNTN--LFLHDDQSGRTV.NTNLINQINE--ETLVAE.LDN..FVLKYSNGK---S
3. La_Lal2_a4      KRLPATV-V.IA..DHPLSNSTAT.KISNTN--LFLDDEQSGPPV.KTNLINHINE--EPLVAE.LDN..FVLRYSNRK---S
4. Al_Lal2         KRFTTRV-V.VA..DDPLSSSIGT.KVDNSN--IILLD-QSGGVA.TTSLTKNMIN-NQLLVAK.LDN..FVLRFSNSS---S
5. Carubv10025960m KRFPEAV-V.VA..DNHLHNSTAT.IFSSSSSTLKLVD-QSGGVV.TSQL-RNRIN-NQRIVPE.LDN..FVFREQLAA---G
6. Bra010990       RQFPGRV-V.TG..GSPLYSSEGK.QISSSAG-IQLFD-ESGYMT.HRDLTSPAAEDDAPLSAY.SDT..FIVSNYSG----G
7. Al_SRK14        KTISIRTYV.VA..DHPLYSSAGT.KISGIN--LVLLN-QSNIAV.STNLTGA-VR--SPPVAE.LPN..FVLRYSKTNGQDI
8. Ah_SRK28        KTISVRTYV.VA..DHPISSSDGT.KISGIN--LVLLN-QSNITV.STNLTGA-VR--SPVVAE.LPN..FVLRNSKTNGHDV
9. Al_SRK01        KKIPERTYV.VA..DTPLSNSVGT.KISDGN--LVILD-HSNIPI.STN-TKGDVR--SPIVAE.LDT..LVIRYFNNN-SQE
10. Al_SRK25       KNVSEKTYV.VA..DKPLSNSIGI.KITNAN--LVLLN-HYDTPV.STNLTGA-VR--SPVVAE.HDN..FVLRDSKTNASDR
11. Ah_SRK13       KEIPKRTYV.VA..DNPLSNSTGT.KISDNN--LVLVD-QFNTLV.STNVTGA-VR--SLVVAE.LAN..LVLRDSKINETDG
12. Ah_SRK43       KTILQRTYV.VA..DKPLINPIGT.KISNTN--LVLLD-SSDTLV.STNLTERDVI--SPVVAQ.LDN..FVLRYSNKDVQSE
13. Bo_SRK12       KKLPYRTYI.VA..DNPLSNSTGT.KISGSN--LVILG-HSNKSV.STNLTRGNER--SPVVAE.LAN..FVMRDSNNNDASK
14. Br_SRK54       KKVYFRTYV.VA..DNPLSRSIGT.RISNMN--LVLLD-HSNKSV.STNLTRGNER--SPVVAE.LAN..FVMRDSNNNDASG
15. Br_SRK60       KKVSQKTYA.VA..DNPLSNSIGT.KISGNN--LVLLG-QSNNTV.STNLTRENVR--SPVIAE.LPN..FVMRYSNNKDSSG
16. At_ARK1        KIIPIRTYV.VA..DNPLSSSNGT.KISGNN--LVIFD-QSDRPV.STNITGGDVR--SPVAAE.LDN..FLLRDSNNR----
17. At_ARK3        KAISKRTYV.VA..DTPLSSSIGT.KISDSN--LVVLD-QSDTPV.STNLTGGDVR--SPLVAE.LDN..FVLRDSKNSAPDG
                   [B-lectin (SM00108)]
                      [B-lectin Pfam (PF01453)]

                      170       180       190       200       210       220       230       240
Consensus          XXWXSFXXPXBXLJXXMXJGXXXXXXXXXXXJXXXXXXXXXXXXXXXXXXXXXXXXXXXXXXXXXXXXXXXRXXXXXXXXX
1. La_Lal2_a1-1    FQ.Q..DY.TDF.LPG.KL.WDRTKNLNKT-LTSWASLTEPTSGSYVFAIENW-TVSHGLLYSN----GQLEF.TG-PLY---
2. La_Lal2_a2      SL.Q..DY.TDT.LPG.KL.LDRTKNLNKT-LTAWASLYDPSSGSYVFKIENW-KVSHGLLYDT----GQIDS.TG-PSY---
3. La_Lal2_a4      FL.Q..DY.TDT.LPG.KL.WDRTKNLNKT-LTSWASLYDPSSGNYDFEIENW-KVSHGLITNT----GQPEF.TD-LSY---
4. Al_Lal2         YL.Q..DF.TDT.LPG.KL.WDRRTNHTKS-LISWNSSDDPSSGRYVYKIDTL-KPSQGLIIFG---DDLPVS.PG-PSY---
5. Carubv10025960m FL.E..DS.TDT.LPG.KL.WDRRTNVNTTSLRSWKSLYDPSYGDYKFQVEIW-ELSQGFIWKN--EDMYLQS.IGLSNH---
6. Bra010990       IL.G..DY.SNV.IPG.VL.YYPGLDYIRT-IT-YDDIFHEGGTETGYEHYIW-GSS-----------GTKIC.ID-PIYTTK
7. Al_SRK14        LL.Q..DY.TDT.LPH.KL.LDLKTGNNRL-LTSWKNSFDPSSGYISYKLETL-GLPEFFMWRN----EVPIF.SG-PWDGTR
8. Ah_SRK28        FM.Q..DN.TDT.LPH.KL.LDLKTGNNRF-LTSWKNAYDPSSGYLSYKLEMQ-GLPEFLMLRG----GGPVF.SG-PWDGFR
9. Al_SRK01        FL.Q..DF.TDT.LPE.KL.WDRKTGLNRF-LRSYKSSNDPTSGSFSYKLETG-VYSEFFMLAK----NSPVY.TG-PWNGIQ
10. Al_SRK25       FL.Q..DF.TNT.LPQ.KL.WDHKRGLNRF-LTCWKNSFDPSSGDYMFRLDTQ-GLPEFFGLKN----FLEVY.TG-PWDGHR
11. Ah_SRK13       FL.Q..DF.TDT.LPE.KL.WDLKTGVNKF-LRSWKSPYDPSSGDFSYKLETR-EFPEFFLSWS----NSPVY.SG-PWEGFR
12. Ah_SRK43       FL.Q..HF.TDT.LPQ.KI.LDRKTEFNRF-LRSWRSADDPASGDYSFKLKTR-GVPEFFIWVK---QNTRMY.SG-PWNGIR
13. Bo_SRK12       FS.Q..DY.TDT.LPE.KL.YNLKKGLNRF-LVSWRSSDDPSSGDYSYKLEPR-RLPEFYLLQG----DVREH.SG-PWNGIR
14. Br_SRK54       FL.Q..DF.TDT.LPE.KL.YDLKTGLNRF-LTAWRNSDDPSSGDYSYKLENR-ELPEFYLLKS----GFQVH.SG-PWNGVR
15. Br_SRK60       FL.Q..DF.TDT.LPE.KL.YDFKTGRNRF-LTSWRSYDDPSSGKFTYELDIQTGLPEFILINRFLNQRVVMQ.SG-PWNGIE
16. At_ARK1        LL.Q..DF.TDT.LAE.KL.WDQKTGFNRI-LRSWKTTDDPSSGEFSTKLETS-EFPEFYICSK----ESILY.SG-PWNGMR
17. At_ARK3        VL.Q..DF.TDT.LPE.KL.WDAKTGFNRF-IRSWKSPDDPSSGDFSFKLETE-GFPEIFLWNR----ESRMY.SG-PWNGIR
                   [B-lectin (SM00...]                                     [S_locus_glycop (PF00954)]
                   [B-lectin Pfam (PF01453)]

                   250  ▼    260       270       280       290       300     ▼ 310       320       330
Consensus          XXXXXXXXXXXXXXXXXXXXXXXXXXXXXXXXXXXXXXXXXXXXXXXXXXXXXXXXXXXXXXXXXXXXXXXCBXXXXCXXX
1. La_Lal2_a1-1    -------------RNIVNITETEDEISHSLNITPNVSSTSLLQLTTAGTLQLTEFI--GGERHFLFLFPLD-R.DFYNK.GE-
2. La_Lal2_a2      -------------SNIVNITETEEEISHSLNITTNVGSISLLQMMYTGSLQLLEFI--GGERHILFHFPDG-T.DFYNT.GY-
3. La_Lal2_a4      -------------SNLVNIAETEEEISHSLNITTNVSSISILQLTSAGALRLTELI--GGEKRTLFYFPND-R.DYYNS.GE-
4. Al_Lal2         -------------RKLFNITETDNEITHSLGISTENV--SLLTLSFLGSLELMAW---TGEWNVVWHFPRN-L.DSYGA.GQ-
5. Carubv10025960m -------------DRIFNITESSEEATCTLAMSSNASLHSALRMTFTGSLQLF------VERNLVWSIPFD-Q.DVYDA.GF-
6. Bra010990       -------------AMIQ--TRTTNSYTYSLRRNTTTSYYASLKMSDTGFLIWSEWTRRDRKLKDLVIAPSD-I.DKYTT.GSG
7. Al_SRK14        LSGIPEMQRWKDINISYNFTENKEEVAFTFRVTTPNV-YSRLIMNSEGFLQLSRWNPTLSEWNVFWRSSTS-D.NGYQS.TP-
8. Ah_SRK28        FSGIPEMQNWKFAYIVYNFTENKEDVAFTYRVTTPNF-YAKLTMRFEGFLELSTWDPDMLEWNVFWVSSTA-D.NIYMG.TA-
9. Al_SRK01        FIGMPEMRKSD--YVIYNFTENNEEVSLTFLMTSQNT-YSRLKLSDKGEFERFTWIPTSSQWSLSWSSPKD-Q.DVYDL.GP-
10. Al_SRK25       FSGIPEMQQWD--DIVYNFTENSEEVAYTFRLTDQTL-YSRFTINSVGQLERFTWSPTQQEWNMFWSMPHE-E.DVYGT.GP-
11. Ah_SRK13       FSGMPEMQQWT--NIISNFTENREEIAYTFRDTDQNI-YSRLTMSSSGYLQRFKWISNGEDWNQHWYAPKD-R.DMYKK.GP-
12. Ah_SRK43       FSGMPEMLEFD--YMVYNFTENREEIVYTFLMTNHSI-YSRLTMTPAGYLQQSTWFPTEEEAS--WVSPNE-Q.DTYRI.GP-
13. Bo_SRK12       FSGILEDQKLS--YMVYNFTENSEEVAYTFRMTNNSF-YSRLTLSSTGYFERLTWAPSSVIWNVFWSSPANPQ.DMYRM.GP-
14. Br_SRK54       FSGIPENQKLS--YMVYNFTENSEEVAYTFRMTNNSF-YSRLKVSSDGYLQRLTLIPISIAWNLFWSSPVDIR.DMFRV.GP-
15. Br_SRK60       FSGIPEVQGLN--YMVYNYTENSEEIAYSFQMTNQSI-YSRLTVSDY-TLNRFTRIPPSWGWSLFWSLPTD-V.DSLYF.GS-
16. At_ARK1        FSSVPGTIQVD--YMVYNFTASKEEVTYSYRINKTNL-YSRLYLNSAGLLQRLTWFETTQSWKQLWYSPKD-L.DNYKV.GN-
17. At_ARK3        FSGVPEMQPFE--YMVFNFTTSKEEVTYSFRITKSDV-YSRLSISSSGLLQRFTWIETAQNWNQFWYAPKD-Q.DEYKE.GV-
                   [S_locus_glycop (PF00954)]
```

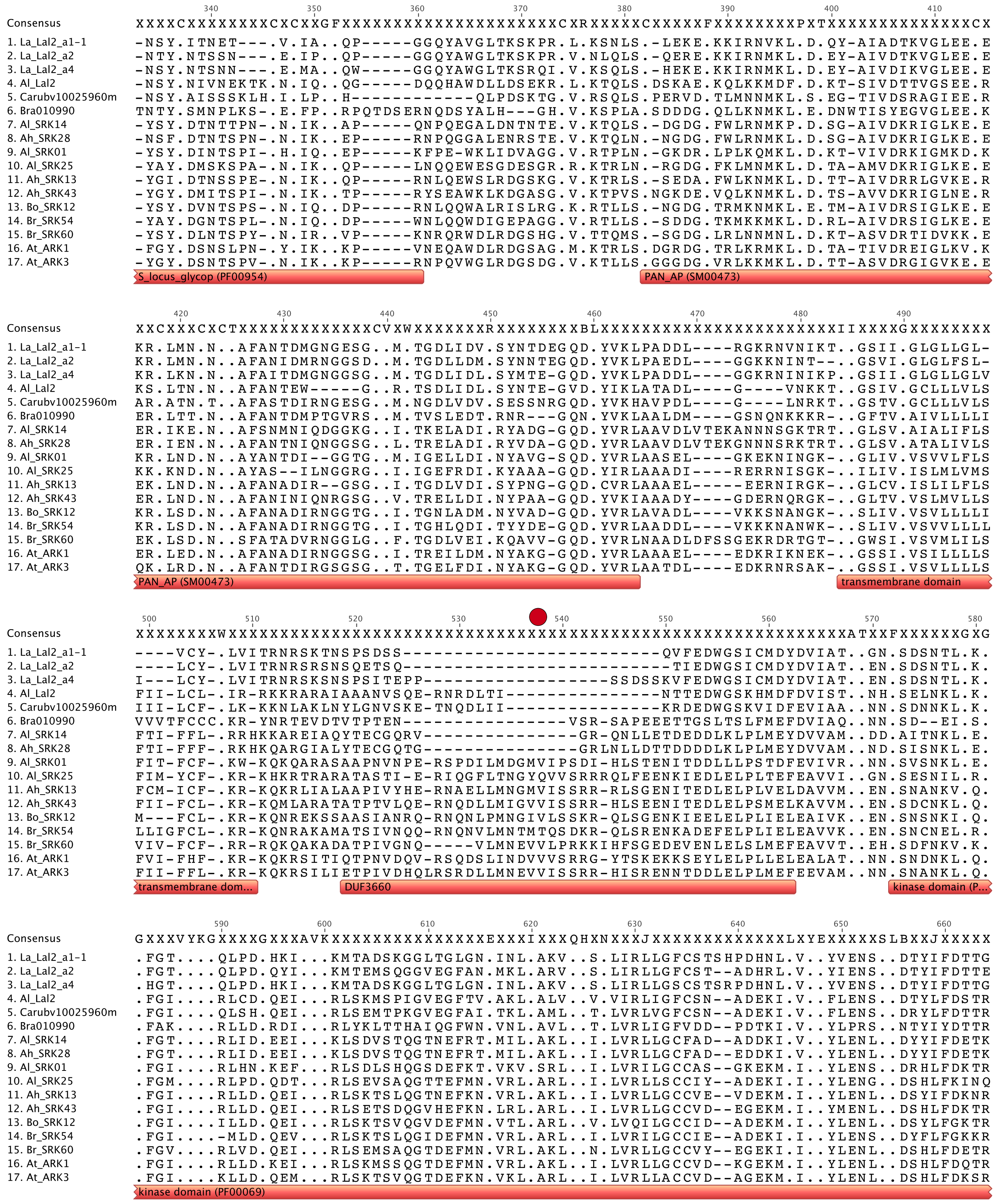

Consensus
1. La_Lal2_a1-1
2. La_Lal2_a2
3. La_Lal2_a4
4. Al_Lal2
5. Carubv10025960m
6. Bra010990
7. Al_SRK14
8. Ah_SRK28
9. Al_SRK01
10. Al_SRK25
11. Ah_SRK13
12. Ah_SRK43
13. Bo_SRK12
14. Br_SRK54
15. Br_SRK60
16. At_ARK1
17. At_ARK3
S_locus_glycop (PF00954)
PAN_AP (SM00473)
transmembrane domain
transmembrane dom...
DUF3660
kinase domain (P...
kinase domain (PF00069)

```
                    670       680       690       700       710       720       730       740
Consensus           XXXLBWXXRXXIXXGXXXGJXYLHXDSRXXXIHXDLKXXNXLLXXXMXPXISDFGXAXXXXXXXXXXXXXXXXXGTXGYXXPXX

1. La_Lal2_a1-1     QYA.D.EM.FE.IK.IVR.LI...Q...FRI..L...PN.I..DKD.I.K.....L.QTLERNATKGFVETAV..F..IA.EL
2. La_Lal2_a2       QYV.N.EK.FE.IK.IVK.LI...Q...FRI..L...PN.I..DKD.I.K.....L.KILEGNATEGRAPTAV..L..ID.NY
3. La_Lal2_a4       QYV.N.EK.FE.IK.IVR.LI...Q...FRI..L...PN.I..DKK.I.K.....L.QTLEGNATIGFARTAV..F..IA.EL
4. Al_Lal2          GSV.N.DT.FD.AK.IIR.LV...Q...FRI..L...PS.I..GKD.V.K.....M.RILGGDETEAHVTTVT..F..IA.EY
5. Carubv10025960m  GSL.N.EY.MK.TL.IVR.LV...H...FRI..L...PA.V..DKD.S.K.....M.KILGGDETEAHVTTVK..F..IA.EY
6. Bra010990        SDV.D.NK.MD.AK.IAR.LL...Q...VRI..L...LS.V..CDQ.I.R.....T.KRLDGEDTEVVASSAT..Y..MA.EY
7. Al_SRK14         SSD.N.QT.FN.IN.IAR.LL...K...CKV..R...TS.I..DKD.I.K.....L.RIFARDEEEATTRRIV..Y..MA.EY
8. Ah_SRK28         SSE.N.QT.FN.IN.IAR.LL...K...CKV..R...TS.I..DKD.I.K.....L.RIFARDEEEATTRRIV..Y..MA.EY
9. Al_SRK01         SSN.N.QR.FD.TN.IAR.IL...H...CRI..R...AS.I..DKN.I.K.....M.RIFSDDVNEAITRRIV..Y..MS.EY
10. Al_SRK25        SSK.N.QK.FN.IN.IAR.LL...Q...FKI..R...AS.V..DKN.T.K.....M.RIFERDETEANTRKVV..Y..MS.EY
11. Ah_SRK13        SWK.N.QM.FN.TN.IAR.LL...Q...CRI..R...AS.V..DKD.T.K.....M.RIFGREETEANTKKVV..Y..MS.EY
12. Ah_SRK43        SCK.N.QM.FD.TT.IAR.IL...Q...CRI..R...AS.V..DKD.T.K.....M.RIFGREETEANTRKVV..Y..MS.EY
13. Bo_SRK12        RSK.N.KE.FD.TN.VAR.LL...Q...FRI..R...VS.I..DRN.V.K.....M.RIFARDETEANTMKVV..Y..MS.EY
14. Br_SRK54        SSN.N.KD.FA.TN.VAR.LL...Q...FRI..R...PG.I..DKY.I.K.....M.RIFARDETQVRTDNAV..Y..MS.EY
15. Br_SRK60        SCM.N.QM.FD.IN.IAR.LL...Q...FRI..R...AS.V..DKD.T.K.....M.RIFGQDETEADTRKVV..Y..MS.EY
16. At_ARK1         SSN.N.QK.FD.IN.IAR.LL...Q...CRI..R...AS.V..DKN.T.K.....M.RIFGREETEANTRRVV..Y..MS.EY
17. At_ARK3         NSK.N.QM.FD.IN.IAR.LL...Q...FRI..R...AS.I..DKY.T.K.....M.RIFGRDETEANTRKVV..Y..MS.EY
                    kinase domain (PF00069)

                    750       760       770       780       790       800       810       820       830
Consensus           XXXXXXSXKXDVXSFGVXXLEXXXXXXXXXXXXXXXXXXXXXXWXXXXXXXXXXXXXDXXXXXXXXXXXXXXXXXXXZXXXCXXX

1. La_Lal2_a1-1     RNDNVY.V.S..Y....ML..IVSGKKNMEYFKNFDGTSLLRYI.DSWSKGKVLEIV.PVLKDSSLSS---LQEEEIRR.VQI
2. La_Lal2_a2       SKHNIY.A.S..Y....LL..IVSGKRNMDFLNSFDGTSLLTHI.NSWSKGEVLEIV.PVLKIASLTS---LQAEEILK.VHI
3. La_Lal2_a4       RNDNVY.V.S..Y....ML..IVSGKKNMEFIKNFDGTSLLRII.DSWSKGEVLEIV.PVLKDSSLSS---LQEEEIRR.VQI
4. Al_Lal2          RSDGVL.V.S..F....ML..IISGKRNIDFLHLNDGSTLLSYM.NHWSQGNGLEIV.PAIKDSSSSS------QQILR.VQI
5. Carubv10025960m  RNDGTI.V.S..F....MV..IVSGKRNIDFLNLDDGSTLLSYI.QRWSEGNGLEIV.PAIKDSSSSV---FP--QVLR.IQI
6. Bra010990        AIDGVC.V.A..F....LL..MVSGINAREFYWKNDYKSFVGFM.NLWLQGKVLDIV.PYFTSSSSSS-SSYQPEEALR.IQI
7. Al_SRK14         AMDGVY.E.S..F....VI..IVTGKKNRGFTSSDLDTNLLSYV.RNMEEGTGYKLL.PNMMDSSSQA---FKLDEILR.ITI
8. Ah_SRK28         AMDGVY.E.S..F....VI..IVTGKKNRGFTSSDLDTNLLSYV.RNMEEGTGYKLL.PNMIDSSSQA---FKLDEILR.ITI
9. Al_SRK01         AMDGIY.E.S..F....ML..IVTGMKNRGFFNSDLDSNLLSYV.RNMEEEKGLAVA.PNIIDSSSLS-PTFRPDEVLR.IKI
10. Al_SRK25        AMDGIF.V.S..F....LV..IISGKRNRGFYNSNQDNNLLSYT.DNWKEGEGLKIV.PIIIDSSSSF-SMFRPYEVLR.IQI
11. Ah_SRK13        AMDGVF.M.S..F....LL..IISGKRNKGFYNSDNDLNLLGCV.RNWTEGKGLEIV.PIILESSSST---VILQEILK.MQI
12. Ah_SRK43        AMDGIF.M.S..F....LL..IISGKRNKGFYNSNGDLNLLGFV.RNWKEGKWTEII.PAIIDSSSSSLSDFQPQEVLR.IQV
13. Bo_SRK12        AMGGIF.E.S..F....MV..IITGKRNRGFY----EDNLLSYA.RNWKGGRALEIV.PVIVNSFSPLSSTFQLQEVLK.IQI
14. Br_SRK54        AMYGVI.E.T..F....IV..IVIGKRNRGFYQVNPENNLPSYA.THWAEGRALEIV.PVILDSLSSLPSTFKPKEVLK.IQI
15. Br_SRK60        AMNGTF.M.S..F....LL..IISGKRNKGFCDSDSNLNLLGCV.RNWKEGQGLEIV.RVIIDSSSPT---FRPREILR.LQI
16. At_ARK1         AMDGIF.M.S..F....LL..IISGKRNKGFYNSNRDLNLLGFV.RHWKEGNELEIV.PINIDSLSSK---FPTHEILR.IQI
17. At_ARK3         AMDGIF.M.S..F....LL..IISSKRNKGFYNSDRDLNLLGCV.RNWKEGKGLEII.PIITDSSST----FRQHEILR.IQI
                    kinase domain (PF00069)

                          840       850       860       870       880       890       900 904
Consensus           XLXCXXXXXXEXRPXMXXXXXXLXXXXXXXXXXXXPXXXXXXXXXXXXXXXXXXXXXXXXXXTXXXXXXXXXXXXXR*

1. La_Lal2_a1-1     G.L.VHESP.D..T.TLILSL.GKEVDFIDRPKP.----------AETEWTGIKGEASTS.APPIAST-MGA..
2. La_Lal2_a2       G.L.VHELP.D..T.SLVGSL.GKEVDFIDRPKP.----------AEIGSKEAKGEASTV.SPQITFS-MDA..
3. La_Lal2_a4       G.L.VHESP.D..T.TLVLSL.GKEVDFIDRPKP.----------AETEWTGIKGEASTS.APPIGST-MGA..
4. Al_Lal2          G.M.VQELP.D..T.SSVGLM.GRETEAIPQPKS.--------VETGSSSGGQQ-ESESG.VPEITLF-IEG..
5. Carubv10025960m  G.L.VQPLP.D..T.SAVGLM.AREAEVIPLPRS.--------VEIGSSSRGGQEESVSG.VPDITMF-IEC..
6. Bra010990        G.L.VQAHR.D..P.ASIILM.GSQNELISLPKP.ADLL----LLQDP-------QGESF.ASVATG*
7. Al_SRK14         G.T.VQEYA.D..M.SWVVSM.GSNTD-IPKPKP.GYCLA---ISSDPWTS---------.TIEYTTTEVEP..
8. Ah_SRK28         G.T.VQEYA.D..M.SWVVSM.GSDTD-IPKPKP.GYCLA---ISSDPWTS---------.TIEYTTTEVEP..
9. Al_SRK01         A.L.VQEYA.D..T.LSVVSM.GSETAEIPKAKA.GYCVGRSLHDTDFSSS------LTW.FG-FAFSDIEP..
10. Al_SRK25        G.L.VQERA.D..K.SSVVLM.GSEKGDIPQPKP.GYCVGRSSLETDSSSSTQR-GDESL.VNQITLSVING..
11. Ah_SRK13        G.L.VQERA.D..R.SSVVAM.GSETAVVPQPKL.GYCVGRSPLETDSSRSKQH-DDESW.VNEITLSVIDA..
12. Ah_SRK43        G.L.VQERA.E..T.SSVVVM.GSETAAIPHPKP.GYCVGRNLLETVSSSS-----DESC.VNQITISIMDA..
13. Bo_SRK12        G.L.VQELA.N..T.SSVVWM.GNEATEIPQPKS.G-CVKRSPYELDPSSSRQRDDDESW.VNQYTCSVIDA..
14. Br_SRK54        G.L.IQERA.H..T.SSVVWM.GSEATEIPQPKP.VYCLIASYYANNPSSSRQFDDDESW.VNKYTCSVIDA..
15. Br_SRK60        G.L.VQERV.D..M.SSVVLM.GSETALIPQPKQ.GYCVSQSSLETYSSWSKLR-DDENW.VNQITMSIIDA..
16. At_ARK1         G.L.VQERA.D..V.SSVMVM.GSETTAIPQPKR.GFCIGRSPLEADSSSSTQR-DDEC-.VNQITLSVIDA.
17. At_ARK3         G.L.VQERA.D..T.SLVILM.GSESTTIPQPKA.GYCLERSLLDTDSSSSKQR-DDESW.VNQITVSVLDA.
                    kinase domain (PF00069)  DUF3403 (PF11883)
```
