# Supplementary figures and images for: Secondary Evolution of a Self-Incompatibility Locus in the Brassicaceae Genus Leavenworthia
Source: PLoS Biol. 2013 May 14;11(5):e1001560. doi: 10.1371/journal.pbio.1001560 (PMC3653793; doi:10.1371/journal.pbio.1001560)

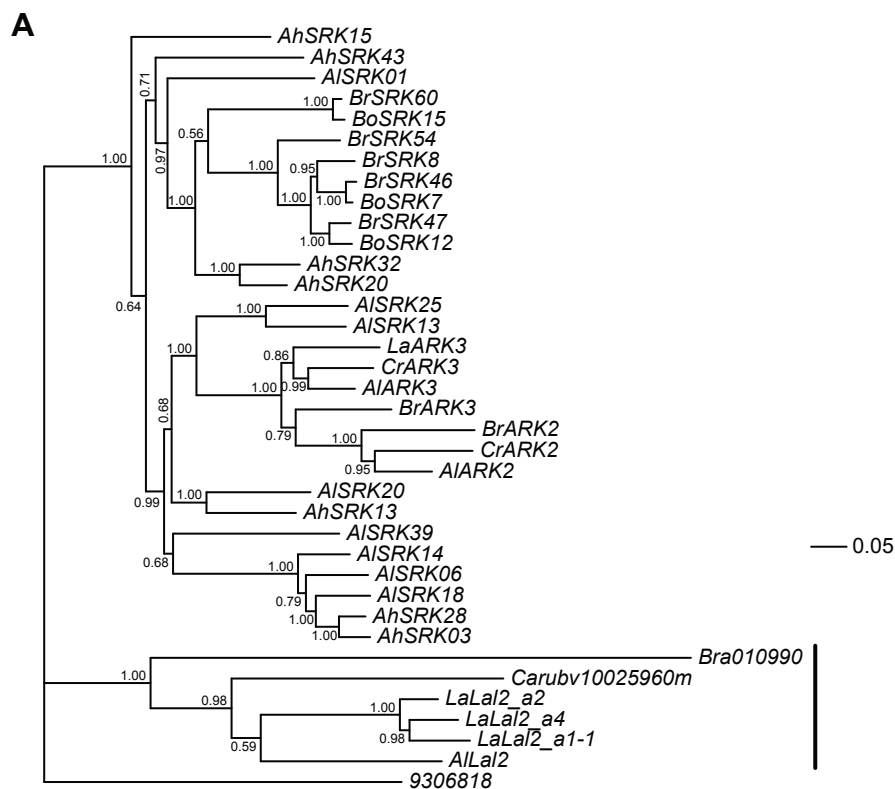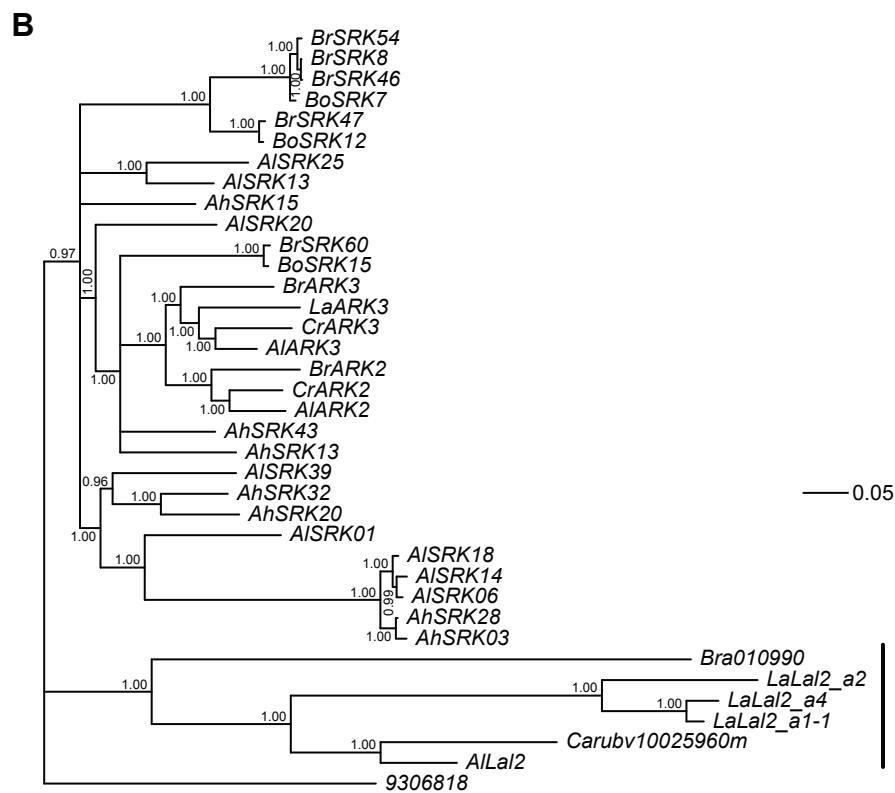

Supplement: Figure S3 — Phylogenetic reconstruction of the relationships among Lal2, Lal2-like, ARK, and SRK for different portions of the sequence. Bayesian 50% consensus phylogeny for the S-domain (A) and the transmembrane and kinase domains (B) of Lal2, Lal2-like, ARK, and SRK sequences used in this study. Posterior probabilities for each bifurcation are indicated at the nodes. Lal2 sequences form a clade separate and distinct from ARK and SRK sequences (vertical bars). The outgroup in each tree is identified by its NCBI gene ID number. (PDF) [file pbio.1001560.s003.pdf]

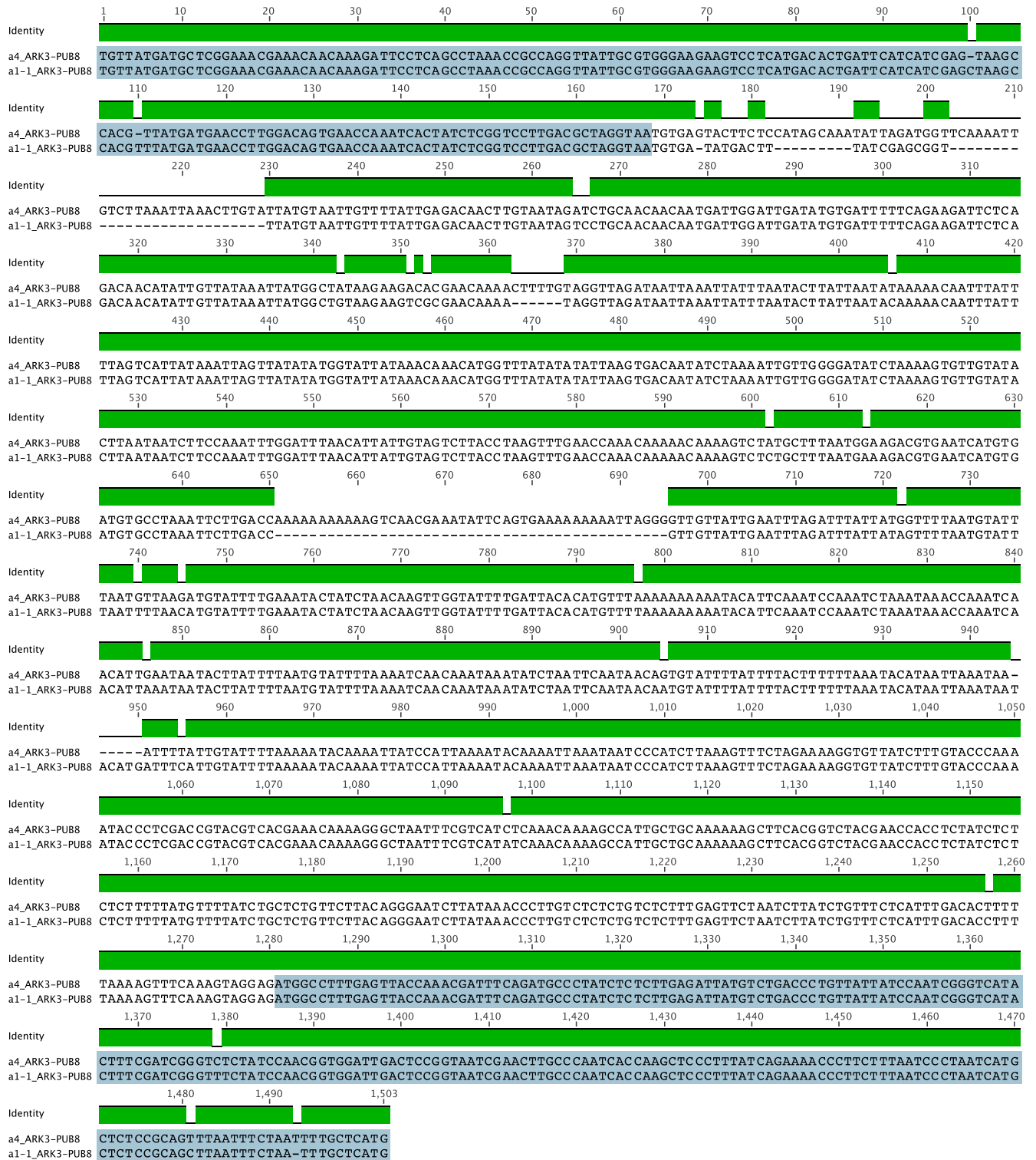

Supplement: Figure S4 — Sequence alignment of the ARK3-PUB8 intergenic region in Leavenworthia SC a4 and SI a1-1 plants. Highlighted in blue are the 3′ end of the coding sequence of ARK3 (top) and the 5′ end of the PUB8 (bottom) orthologs. The a4 sequence was extracted from Leavenworthia scaffold 2269 (Figure 6A). The a1-1 sequences were obtained by PCR amplification using primers anchored in the ARK3 and PUB8 coding sequences, followed by end-sequencing of PCR products (size of about 1.5 kb). Note that the a1-1 end sequences obtained do not overlap and the sequence corresponding to a stretch of 45 nt of the a4 sequence (between positions 650 and 696) remains unknown. Green horizontal bars above aligned sequences indicate identity between sequences. The ARK3-PUB8 intergenic regions covered by the a1-1 sequences are 93% identical between a1-1 and a4. (PDF) [file pbio.1001560.s004.pdf]

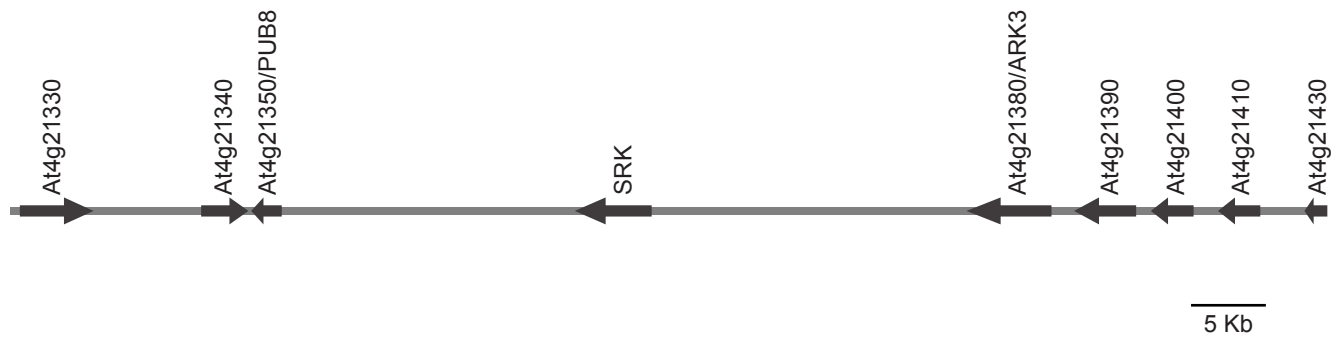

Supplement: Figure S5 — Genomic organization of the S locus in Sisymbrium irio. An SRK gene sequence was identified in a genome region between gene orthologs of A. thaliana PUB8 and ARK3. Genes were annotated using the A. thaliana reference genome. (PDF) [file pbio.1001560.s005.pdf]

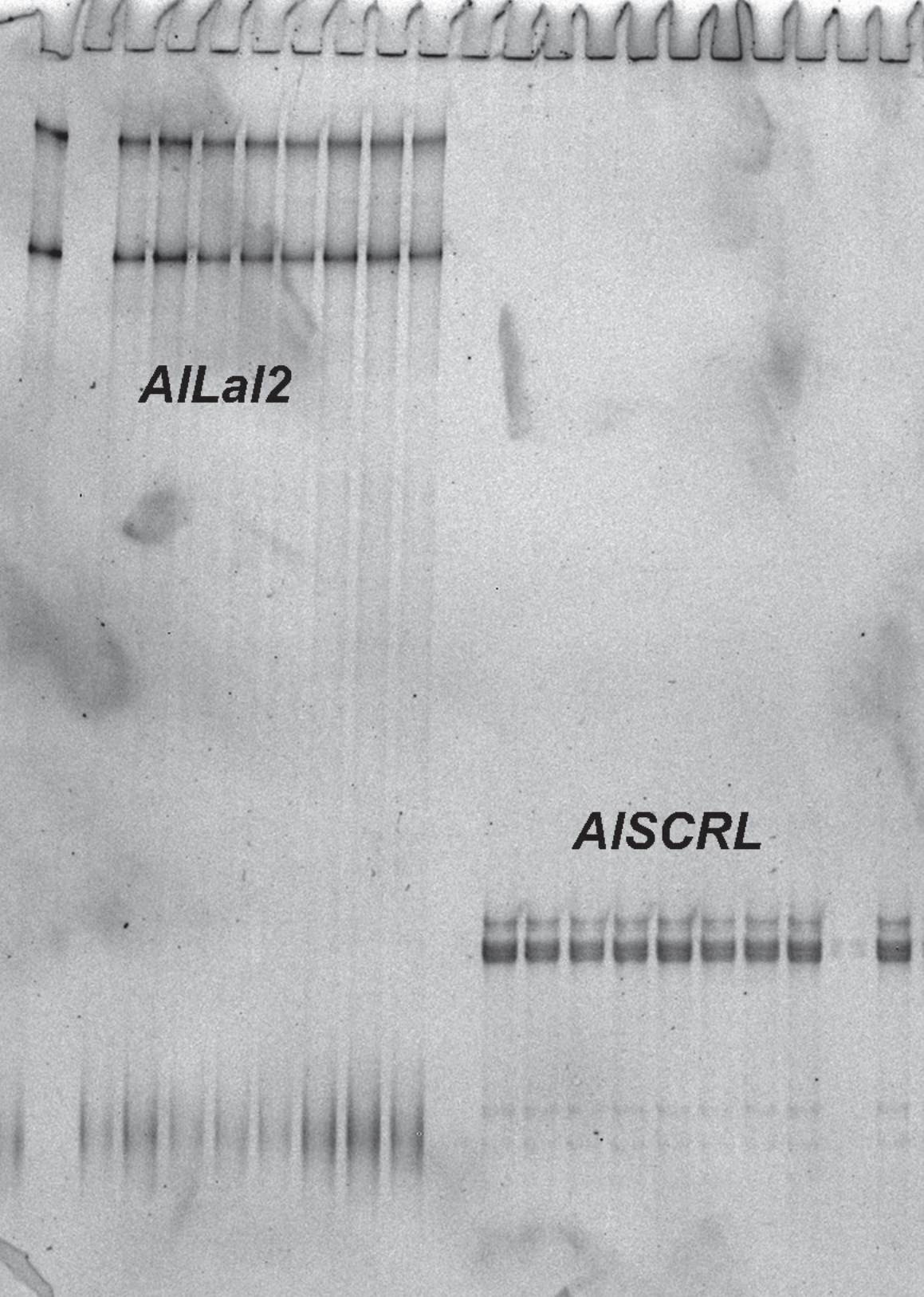

***A/Lal2***

***A/SCRL***

Supplement: Figure S6 — SSCP gel for AlLal2 and AlSCRL from 10 individuals from a single A. lyrata population. The observed banding patterns indicate monomorphism for both loci (see text for details). (PDF) [file pbio.1001560.s006.pdf]
