# Supplementary material for: Secondary Evolution of a Self-Incompatibility Locus in the Brassicaceae Genus Leavenworthia
Source: PLoS Biol. 2013 May 14;11(5):e1001560. doi: 10.1371/journal.pbio.1001560 (PMC3653793; doi:10.1371/journal.pbio.1001560)
Supplement: Figure S7 — Alignment of the a2 full-length and a1-2 partial LaLal2 amino acid sequences. The a1-2 aa sequence was deduced from cDNA sequence obtained by using primers anchored in exon 1 and exon 7 of the gene (see Table S5 for primer sequences) and corresponds to positions 169 to 714 of the a2 LaLal2 aa sequence. Green horizontal bars above aligned sequences represent identity between sequences. Note that the available aa sequence of a1-2 is identical to that of a2 except for one amino acid residue located in the intracellular kinase domain. The predicted transmembrane domain is highlighted with a blue box to delimit the extracellular domain versus the intracellular domain. (PDF) [file pbio.1001560.s007.pdf]

```
        1       10        20        30        40        50        60        70        80        90
a2_Lal2 MTTHNNSYTFFPLFLLVISFILRMSINGFSLTARESVKLSEDTRNIVSPGEIFEMGLFKAATSLTDIDGWYLGIWYKQLPRIVVWIANRD

                100       110       120       130       140       150       160       170       180
Identity
a2_Lal2    SHLSNSTATLKMSNTNLFLHDDQSGRTVWNTNLINQINEETLVAELLDNGNFVLKYSNGKSSLWQSFDYPTDTLLPGMKLGLDRTKNLNK
a1-2_Lal2                                                                                KLGLDRTKNLNK

                190       200       210       220       230       240       250       260       270
Identity
a2_Lal2    TLTAWASLYDPSSGSYVFKIENWKVSHGLLYDTGQIDSRTGPSYSNIVNITETEEEISHSLNITTNVGSISLLQMMYTGSLQLLEFIGGE
a1-2_Lal2  TLTAWASLYDPSSGSYVFKIENWKVSHGLLYDTGQIDSRTGPSYSNIVNITETEEEISHSLNITTNVGSISLLQMMYTGSLQLLEFIGGE

                280       290       300       310       320       330       340       350       360
Identity
a2_Lal2    RHILFHFPDGTCDFYNTCGYNTYCNTSSNCECIPGFQPGGQYAWGLTKSKPRCVRNLQLSCQEREFKKIRNMKLPDTEYAIVDTKVGLEE
a1-2_Lal2  RHILFHFPDGTCDFYNTCGYNTYCNTSSNCECIPGFQPGGQYAWGLTKSKPRCVRNLQLSCQEREFKKIRNMKLPDTEYAIVDTKVGLEE

                370       380       390       400       410       420       430       440       450
Identity
a2_Lal2    CEKRCLMNCNCTAFANIDMRNGGSDCVMWTGDLLDMRSYNNTEGQDLYVKLPAEDLGGKKNINTIIGSVIGGLGLFSLLCYWLVITRNRS
a1-2_Lal2  CEKRCLMNCNCTAFANIDMRNGGSDCVMWTGDLLDMRSYNNTEGQDLYVKLPAEDLGGKKNINTIIGSVIGGLGLFSLLCYWLVITRNRS

                460       470       480       490       500       510       520       530       540
Identity
a2_Lal2    RSNSQETSQTIEDWGSICMDYDVIATATENFSDSNTLGKGGFGTVYKGQLPDGQYIAVKKMTEMSQGGVEGFANEMKLIARVQHSNLIRL
a1-2_Lal2  RSNSQETSQTIEDWGSICMDYDVIATATENFSDSNTLGKGGFGTVYKGQLPDGQYIAVKKMTEMSQGGVEGFANEMKLIARVQHSNLIRL

                550       560       570       580       590       600       610       620       630
Identity
a2_Lal2    LGFCSTADHRLLVYEYIENSSLDTYIFDTTEQYVLNWEKRFEIIKGIVKGLIYLHQDSRFRIIHLDLKPNNILLDKDMIPKISDFGLAKI
a1-2_Lal2  LGFCSTADHRLLVYEYIENSSLDTYIFDTTEQYVLNWEKRFEIIKGIVKGLIYLHQDSRFRIIHLDLKPNNILLDKDMIPKISDFGLAKI

                640       650       660       670       680       690       700       710       720
Identity
a2_Lal2    LEGNATEGRAPTAVGTLGYIDPNYSKHNIYSAKSDVYSFGVLLLEIVSGKRNMDFLNSFDGTSLLTHIWNSWSKGEVLEIVDPVLKIASL
a1-2_Lal2  LEGNATEGHAPTAVGTLGYIDPNYSKHNIYSAKSDVYSFGVLLLEIVSGKRNMDFLNSFDGTSLLTHIWNSWSKGEVLEIVDPV

                730       740       750       760       770       780       790    797
a2_Lal2    TSLQAEEILKCVHIGLLCVHELPEDRPTMSLVGSLLGKEVDFIDRPKPPAEIGSKEAKGEASTVTSPQITFSMDAR*
```
