# Supplementary material for: Secondary Evolution of a Self-Incompatibility Locus in the Brassicaceae Genus Leavenworthia
Source: PLoS Biol. 2013 May 14;11(5):e1001560. doi: 10.1371/journal.pbio.1001560 (PMC3653793; doi:10.1371/journal.pbio.1001560)
Supplement: Table S2 — Estimates of the ratio and rates of nonsynonymous and synonymous substitution per site for four major protein domains in a comparison of Lal2 and SRK coding sequences. (DOCX) [file pbio.1001560.s009.docx]

Table S2. Estimates of the ratio and rates of non-synonymous and synonymous substitution per site for four major protein domains in a comparison of *Lal2* and *SRK* coding sequences^1^ ________________________________________________________________

| Domain | dN/dS | dN | dS | Nucleotides |
| --- | --- | --- | --- | --- |
| B-lectin | 0.3293 | 0.3726 | 1.1312 | 450 |
| S_locus_glycoprotein | 0.2581 | 0.6138 | 2.3781 | 291 |
| Pan_Apple | 0.2062 | 0.2413 | 1.1701 | 240 |
| Kinase | 0.2106 | 0.3368 | 1.5989 | 849 |
| Domain | B-lectin | S-locus glycoprotein | Pan-Apple | Kinase |
| B-lectin | _ | 0.9724 | 3.3746 | 3.0970 |
| S_locus_glycoprotein | 0.07129 | _ | 0.2293 | 0.1911 |
| Pan_Apple | 0.12317 | 0.0518 | _ | 0.0032 |
| Kinase | 0.11875 | 0.0475 | 0.0044 | _ |

^1^Sequences compared are *LaLal2* (a1-1 haplotype) and *Arabidopsis halleri* *SRK15*. Maximum likelihood estimates of parameters obtained using the PAML package program CODEML [87]. In the matrix portion of the table (below the estimates), the upper diagonal gives the log likelihood ratio test statistic value when dN/dS ratios are constrained to be equal for the comparison denoted in each cell. The lower diagonal gives the absolute value of the difference between the dN/dS ratios for the comparison denoted in each cell. The test statistic is distributed as Chi square with 1 degree of freedom. None of the pairwise comparisons are statistically significant.
