# Supplementary material for: Secondary Evolution of a Self-Incompatibility Locus in the Brassicaceae Genus Leavenworthia
Source: PLoS Biol. 2013 May 14;11(5):e1001560. doi: 10.1371/journal.pbio.1001560 (PMC3653793; doi:10.1371/journal.pbio.1001560)
Supplement: Table S4 — RNAseq expression analysis of AlLal2, AlSCRL, SRK, and SCR in Arabidopsis lyrata strain MN47. (DOCX) [file pbio.1001560.s011.docx]

Table S4. RNAseq expression analysis of *AlLal2*, *AlSCRL*, *SRK* and *SCR* in *Arabidopsis lyrata* strain MN47*

|  | *AlLal2* | *AlSCRL* | *SRK* | *SCR* |
| --- | --- | --- | --- | --- |
| root | 0 | 0 | 0 | 0 |
| flower bud  (stage 12) | 0.292963612 | 28.98805663 | 3.820121805 | 580.0137928 |
| seedling | 0 | 0 | 0.214032795 | 0 |

*Cells values in table are in units of fragments per kilobase of exon per million fragments mapped (FPKM). Library sizes are as follows: root (34 x 10^6^ reads). flower bud (25.3 x 10^6^ reads), seedling (26.2 x 10^6^ reads).
